# Systematic mutagenesis reveals dominant–minor paralog configurations in the rice *GA2ox* gene family

**DOI:** 10.3389/fpls.2026.1813123

**Published:** 2026-05-07

**Authors:** Kun-Ting Hsieh, Shiau-Yu Shiue, Yi-Chun Liao, I-Wen Wang, Ching-Shan Tseng, Wen-Hsiung Li, Liang-Jwu Chen

**Affiliations:** 1 Institute of Molecular Biology, National Chung Hsing University, Taichung, Taiwan; 2 Biodiversity Research Center, Academia Sinica, Taipei, Taiwan; 3 Division of Biotechnology, Taiwan Agriculture Research Institute, Taichung, Taiwan; 4 Department of Ecology and Evolution, University of Chicago, Chicago, IL, United States

**Keywords:** CRISPR-induced variants, GA homeostasis, gibberellin 2-oxidases, paralog configurations, plant height regulation, rice

## Abstract

**Introduction:**

Gibberellin 2-oxidases (GA2oxs) deactivate bioactive gibberellins and play essential roles in regulating rice growth and development. However, the relative contributions of individual *OsGA2ox* paralogs, their redundancy patterns, and dominant–minor relationships within this multigene family remain poorly understood. Clarifying these relationships is important for understanding how GA2ox genes coordinately influences plant development in rice.

**Methods:**

To address these questions, we generated 36 CRISPR/Cas9-edited rice lines targeting all nine *OsGA2ox* genes, including single-, double-, triple-, and quadruple-gene knockout mutants, and systematically analyzed their effects on agronomic traits to compare gene-specific and combinatorial functions.

**Results:**

In Class II, *OsGA2ox1* is the main regulator of plant height, whereas *OsGA2ox2* alone showed no discernable effect. Double mutants of these two genes showed a modest height increase and a reduced grain number but retained normal flowering time, indicating partial redundancy in regulating plant height and grain number, with *OsGA2ox1* as the dominant contributor. In Class I, *OsGA2ox3*, *OsGA2ox4*, and *OsGA2ox8* function as dominant suppressors of stem elongation, whereas *OsGA2ox7* showed no phenotypical effect in single mutants but contributed to stem elongation control when disrupted together with other Class I members. The quadruple Class I mutant exhibited the greatest increase in plant height but reduced grain weight and fertility. In Class III, *OsGA2ox5* and *OsGA2ox6* regulated plant height, with *OsGA2ox6* additionally influencing grain size, whereas *OsGA2ox9* showed no detectable function.

**Discussion:**

These findings reveal a hierarchical but partially redundant regulatory framework within the rice GA2ox gene family, in which dominant and minor paralogs contribute unequally to GA-related growth regulation. Our study provides allele-validated genetic evidence for functional diversification among *OsGA2ox* paralogs and highlights how redundancy and specialization are integrated to stabilize hormone-regulated development and key agronomic traits in rice.

## Highlight

- Systematic mutagenesis of all rice GA2ox genes reveals dominant–minor paralog configurations that modulate stem elongation, flowering time, and yield via gibberellin homeostasis.

## 1 Introduction

Gibberellins (GAs) are plant diterpenoid hormones comprising more than 130 known structures, yet only GA_1_, GA_3_, GA_4_, and GA_7_ function as the principal bioactive forms in higher plants (Yamaguchi, 2008). These hormones regulate diverse developmental processes, including germination, vegetative growth, flowering and grain development (Robil et al., 2025). To support proper growth, the availability of bioactive GAs must be tightly regulated through the coordinated activities of GA biosynthetic and catabolic enzymes.

In plants, three 2-oxoglutarate–dependent dioxygenases (2ODDs) play pivotal roles in GA metabolism (Yamaguchi, 2008): GA 20-oxidases (GA20oxs) and GA 3-oxidases (GA3oxs), which catalyze the final steps of GA biosynthesis, and GA 2-oxidases (GA2oxs), which deactivate bioactive GAs and their intermediates through 2β-hydroxylation to maintain hormonal homeostasis. GA2oxs are widely distributed among seed plants and form multigene families of variable sizes, typically comprising 8 to 34 members in both eudicots and monocots (Yamaguchi, 2008; Chen et al., 2016; Zhang et al., 2023; Cheng et al., 2024). Phylogenetic analyses classify the GA2oxs into three classes (Lee and Zeevaart, 2005): Class I and Class II, which inactivate C_19_-GAs, and Class III, which acts on C_20_-GAs (Schomburg et al., 2003; Lo et al., 2008). However, the functional distinctions among the three classes, beyond their substrate preferences, remain largely unexplored.

Extensive evolutionary analyses have revealed that the C_19_- and C_20_-GA2ox families emerged in gymnosperms and angiosperms (Yoshida et al., 2020), respectively, and subsequently expanded and diversified independently in eudicots and monocots (Han and Zhu, 2011; Kawai et al., 2014; Takehara et al., 2020; Yoshida et al., 2020). This expansion is thought to have resulted from large-scale genome duplication events (Huang et al., 2015). Such duplications typically preserve cis-regulatory landscapes, giving rise to paralogs with initially overlapping profiles that confer functional redundancy (Almeida-Silva and Van de Peer, 2025). In the case of *GA2ox* genes, whose role is to maintain GA homeostasis, this redundancy likely reflects a need for dosage balance, ensuring stable overall GA catabolic capacity across tissues and developmental stages (Hedden, 2020). However, over evolutionary time, regulatory divergence among duplicated paralogs may have produced asymmetric expression, enabling these paralogs to preserve overall dosage balance (Iohannes and Jackson, 2023). Under this scenario, paralogs may contribute unequally to total GA-catabolic activity, with one acting as the dominant paralog and the other functioning as a minor contributor. Because a dominant–minor configuration has been documented in the *GA20ox* gene family (Plackett et al., 2012), a similar configuration might have occurred among GA2ox paralogs, providing a plausible framework for regulatory divergence in their functional contributions while still maintaining stable GA homeostasis.

The rice genome encodes nine functional GA2ox members, including four class I genes (*OsGA2ox3, OsGA2ox4, OsGA2ox7* and *OsGA2ox8*), two class II genes (*OsGA2ox1* and *OsGA2ox2*), and three class III genes (*OsGA2ox5, OsGA2ox6* and *OsGA2ox9*), with class I further divided into two clades (Hsieh et al., 2021). Functional analyses across mutant lines indicate that *GA2ox* genes contribute to a variety of biological processes. *OsGA2ox1*, which is expressed around the shoot apex, has been proposed to regulate phase transitions (Sakamoto et al., 2001) and is implicated in controlling plant height, grain number, and heading date (Cao et al., 2024). *OsGA2ox3* contributes to GA homeostasis (Sakai et al., 2003), and its knockout leads to elongated internodes and leaves (Takehara et al., 2020). *OsGA2ox4* influences internode elongation and is associated with lodging resistance, with natural amino acid variants linked to plant-height variation among diverse rice varieties (Liu et al., 2018). *OsGA2ox7*, a clade-B class I member, plays a comparatively minor role in vegetative growth, as knockout mutants exhibit only a slight reduction in culm length (Jin et al., 2023). Members of the class III subgroup, *OsGA2ox5, OsGA2ox6*, and *OsGA2ox9*, function predominantly in reproductive development, with individual mutants showing defects in fertility and grain formation without substantial changes in plant height (Chen et al., 2019). Additionally, mutants of *OsGA2ox6* and *OsGA2ox9* display preharvest sprouting, underscoring their roles in regulating seed dormancy (Xing et al., 2023; Shen et al., 2024).

Despite the breadth of these functional characterizations, the relative contributions of individual *GA2ox* genes to overall GA catabolic dosage and whether dominant–minor configurations operate within the rice *GA2ox* network remain not well understood. Although a previous study suggested that paralogs with higher transcript abundance and stronger growth-suppressive effects in overexpression lines may function as dominant contributors to GA catabolism (Hsieh et al., 2021), these inferences remain tentative because overexpression systems do not reflect *in-planta* functions, and therefore cannot reliably establish functional hierarchies within the family.

To directly compare functions *in planta*, we used CRISPR/Cas9 to generate a comprehensive series of single and multiple *osga2ox* knockout mutants for all nine GA2ox genes in *Oryza sativa* cv. Tainung 67 (TNG67). By combining allelic series of single- and multiple-gene mutants within class II, class I, and class III, and by examining a range of agronomic traits, we have systematically assessed the extent of redundancy and functional divergence within the family. This approach enabled us to assess the relative contributions of individual paralogs to GA-catabolic dosage through their phenotypic effects. Our findings reveal a functional hierarchy within and among the rice GA2ox genes and provide a framework for understanding how dominant and minor paralogs cooperate to maintain GA homeostasis and shape key agronomic traits.

## 2 Materials and methods

### 2.1 Plant materials, growth conditions, and agronomic trait investigations

The japonica rice cultivar *Oryza sativa* cv. Tainung 67 (TNG67), which is insensitive to temperature and photoperiod, was used as the wild-type (WT) in this study. Seeds of TNG67 and *osga2ox* variant lines were germinated in a growth chamber at 30 °C under a 16 h light/8 h dark cycle for 14 days and subsequently transplanted to an isolated paddy field at the Taiwan Agricultural Research Institute (TARI).

Agronomic trait analyzed included relative plant height (RPH), heading date, and several yield-related traits, such as panicle number (PN), grain number per panicle (GNP), seed-setting rate (SSR), thousand-grain weight (TGW), and yield per plant (YPP). All traits other than RPH were evaluated with at least three replicates and a sample size of n ≥ 4. Plant height was measured at the yellow-ripening/mature stage. Grain number per panicle was calculated by dividing the total number of seeds per plant by the panicle number per plant. The SSR was determined as the ratio of fertile seeds to total seeds per plant. The TGW was obtained as the weight of 1,000 fertile seeds. Yield per plant was measured as the total weight of fertile seeds from each plant.

### 2.2 Plasmid vector construction and rice transformation

For construction of CRISPR/Cas9 expression vectors, potential sgRNAs targeting the *OsGA2ox* genes were designed using the E-CRISPR tool (Heigwer et al., 2014) (https://www.e-crisp.org/). The sgRNA sequences and their relative positions within the target genes are provided in the **Supplementary Information** (**Supplementary Figures S1A–C**). Selected sgRNA pairs (**Supplementary Table S1**) were synthesized, annealed, and cloned into the *Bsa*I-digested CRISPR/Cas9 vector pRGEB32 (Xie et al., 2015) under the control of the *OsU3* promoter (**Supplementary Figure S1D**). The ligation products were transformed into *E. coli*, and positive clones were verified by *Bsa*I restriction digestion and sequencing. Resulting plasmid constructs were then introduced into *Agrobacterium tumefaciens* strain EHA105 and used for rice transformation following the established protocol (Hiei et al., 1997).

### 2.3 Regeneration of transgenic rice and selection of gene-edited plants

For the selection of T-DNA–containing transgenic plants, callus-derived regenerants were cultured on medium supplemented with 50 mg/L hygromycin. Surviving plants were verified by PCR using primers specific for the hygromycin phosphotransferase (*hpt*) gene and the sgRNA construct (**Supplementary Figure S1D**; **Supplementary Table S1**). Independent line numbers represent plants regenerated from distinct calli.

Gene-edited plants were selected as previously described (Hsieh et al., 2023), and the overall workflow is illustrated in **Supplementary Figure S2-1**. Briefly, the sgRNA-targeted region was PCR amplified from transgenic plants using the appropriate *OsGA2ox* genotyping primer sets (**Supplementary Table S1**). PCR products showing clear insertion/deletion (In/Del) events were subjected to DNA sequencing, and sequences were decoded via CRISPR-ID (Dehairs et al., 2016).

Gene-edited plants were identified in the T0 generation. These plants were further analyzed and classified in the T1 generation as homozygous or heterozygous, either with or without the construct. Homozygous plants (with or without construct) and heterozygous plants without the construct were advanced from the T2 through T6 generations to isolate transgene-free, gene-edited lines. An example of screening for *osga2ox4* mutant variants is shown in **Supplementary Figure S2-2**.

### 2.4 Statistical analysis

All data were analyzed using GraphPad Prism 8 (GraphPad Software Inc., La Jolla, CA, USA) and SPSS (IBM Corp., Armonk, NY, USA). For multiple comparisons, one-way ANOVA followed by Tukey’s honestly significant difference (HSD) test was applied. Comparisons with the wild-type control were performed using Student’s *t*-test. Data are presented as mean ± SD, with the distribution of individual data points shown in the figures. Significant differences are indicated as follows: *p* < 0.05 (*), *p* < 0.01 (**), and *p* < 0.001 (***). In addition, different letters above the bars denote statistically significant differences at *p* < 0.05.

## 3 Results

### 3.1 Generation and selection of knockout mutants for rice *GA2ox* genes

The nine *OsGA2ox* genes, which were classified into three classes (Class I: *OsGA2ox3, 4, 7, 8*; Class II: *OsGA2ox1, 2*; Class III: *OsGA2ox5, 6, 9*), were individually targeted with gene-specific CRISPR/Cas9 vectors (**Supplementary Figure S1**) introduced by Agrobacterium-mediated transformation. A total of 36 single-gene knockout or knockdown variants were successfully generated for the rice *GA2ox* gene family using CRISPR/Cas9-mediated genome editing (**Table 1**).

**Table 1:** Characteristics of the gene-edited variants for rice *GA2ox* family genes and numbers of plant samples used for statistical analysis.

Class/ genes	Lines: variants genotypes	Types of mutation	Resulted activities**	NO. of plants analyzed	T-DNA/ generation
II/ GA2ox1	2.1: +1/+1	Frame shift	KO	26	N / T6
	5.3: -84/-84^#^	28-aa deletion	KD/KO	24	N / T3
	5.4: -42/-42^#^	14-aa deletion	KD/KO	23	N / T3
II/ GA2ox2	2.1: -738/-738	Large deletion	KO	17	Y / T2
	3.1: +1/ +1	Frame shift	KO	13	N / T2
	3.2: -35/-35	Frame shift	KO	13	N / T2
I/ GA2ox3	4.1: +1/+1	Frame shift	KO	8	N / T5
	7.1: -2/-2	Frame shift	KO	8	N / T2
	7.2: -2/+1 ^bi^	Frame shift	KO	13	Y / T2
	8.2: -13/+1^bi^	FS/PMS*	KO	15	Y / T2
	4.1: -3/-3^#^	1-aa deletion	NM	7	N / T5
I/ GA2ox4	1.1: -2/-2	Frame shift	KO	8	N / T6
	2.1: +1/+1	Frame shift	KO	7	N / T2
	3.1: -2/-2	Frame shift	KO	8	N / T2
	3.1: +1/+1	Frame shift	KO	9	N / T2
	10.1: -38/-38	Frame shift	KO	9	N / T2
I/ GA2ox7	5.1: -5/-5	Frame shift	KO	7	N / T2
	6.1: +43/+43	Frame shift	KO	8	N / T2
	8.1: +1/+1	Frame shift	KO	8	N / T2
	10.1: -2/-2	Frame shift	KO	7	N / T2
I/ GA2ox8	1.4: -19/-19	Frame shift	KO	6	N / T2
	2.1: +1/ +1	Frame shift	KO	7	N / T2
	5.1: -41/-41	Frame shift	KO	16	N / T2
	9.1: -33/-33	11-aa deletion	KO/KD	7	Y / T2
III/ GA2ox5	9.1: +1/+1	Frame shift	KO	6	N / T2
	9.2: -7/-7	Frame shift	KO	8	Y / T2
	0.2: -48/-48	16-aa deletion	KO/KD	6	N / T2
	4.1: -12/-12^#^	4-aa deletion	KD/NM	5	N / T2
III/ GA2ox6	1.1: +1/+1	Frame shift	KO	8	N / T5
	1.2: -91/-91	Frame shift	KO	7	N / T5
	4.2: -7/+1^bi^	Frame shift	KO	8	Y / T1
	6.2: -31/+1^bi^	Frame shift	KO	6	Y / T1
III/ GA2ox9	8.1: +25/+25	Frame shift	KO	8	N / T4
	8.1: -2/-2	Frame shift	KO	8	N / T4
	11.1: -5/-5	Frame shift	KO	8	N / T4
	11.2: -55/-21^bi^	FS/ 7-aa deletion*	KO/KD	7	Y / T1

^#^ Samples were not included into its gene for statistical analysis.

^bi^ bi-allelic genotype.

*Different types of mutation in bi-allelic variants. FS, frame shift; PMS premature stop.

**Activities: KO, knock-out; KD, knockdown; NM, normal

Existence of T-DNA construct: Y: yes; N: no. Most T-DNA constructs were crossed out after T2 generation and plants with homozygous genotype without construct can be obtained.

Following each transformation, multiple edited alleles were obtained for a *GA2ox* gene. At least three independent knockout or knockdown variants per gene were identified, with mutations including frameshifts, premature stop codons, and in-frame deletions predicted to abolish or reduce enzyme activity. Detailed indel mutations and their amino acid changes were confirmed (**Supplementary Figures S3-1**–**S3-9**).

To ensure genetic stability, mutant genotypes were tracked across successive generations (T2–T6) (**Supplementary Figure S2**). Except for one *OsGA2ox6* and one *OsGA2ox9* line, which were only obtained in the T1 generation (**Table 1**), all lines were confirmed to be stably inherited. Furthermore, to exclude potential transgene effects, the T-DNA was segregated out by the T2 generation, resulting in 28 transgene-free variant lines, including at least one knockout line for each *OsGA2ox* gene (**Table 1**).

The stable and transgene-free lines, representing all nine *OsGA2ox* genes, were subsequently employed for downstream functional analyses.

### 3.2 Phenotypic assessment of class II *OsGA2ox* genes using single-gene knockout mutants

To evaluate the role of Class II *OsGA2ox* genes, we analyzed knockout (KO) and knockdown (KD) variants of *OsGA2ox1* and *OsGA2ox2*. Three homozygous *osga2ox1* variant lines were obtained (**Table 1**). Among them, the “+1” variant carried a frameshift mutation, generating a KO allele, whereas the “-84” and “-42” variants contained in-frame deletions of 28 and 14 amino acids (**Supplementary Figure S3-1C**), respectively, and were therefore classified as KD lines. For *OsGA2ox2*, three homozygous KO variant lines were identified (**Table 1**). Both the “+1” and “-35” variants carried frameshift mutations, while the “-738” variant carried a large deletion spanning from the second exon to the third exon (**Supplementary Figure S3-2C**).

**Figure 1:**
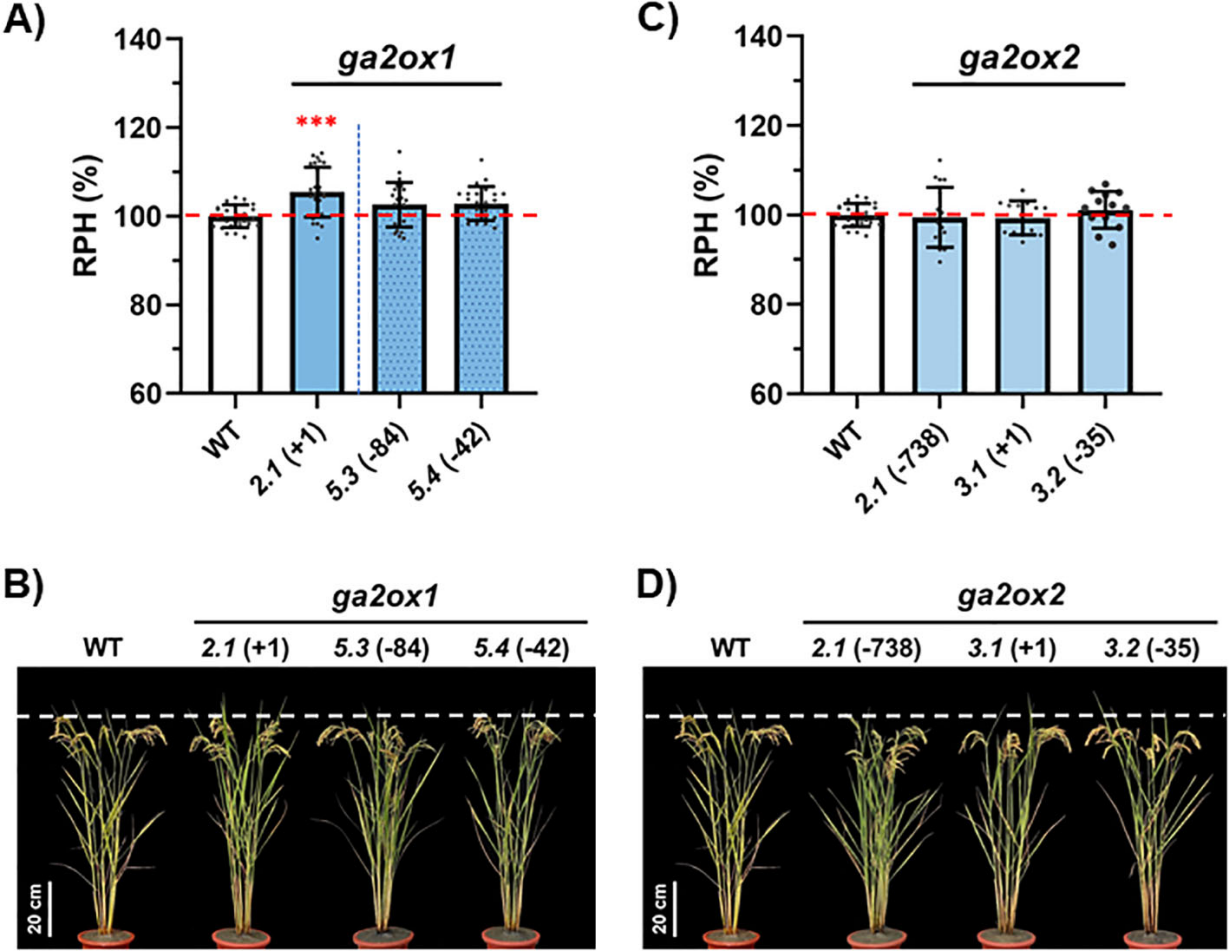
Effects of *osga2ox1* and *osga2ox2* mutations on plant height. **(A, B)** Analysis of *osga2ox1* mutants. **(A)** Relative plant heights (RPH, %) of the wild type (WT) and independent *osga2ox1* mutant lines. **(B)** Representative plants of WT and *osga2ox1* mutants. The genotypes and the line identifiers are shown. **(C, D)** Analysis of *osga2ox2* mutants. **(C)** RPHs of WT and independent *osga2ox2* mutant lines. **(D)** Representative plants of WT and *osga2ox2* mutants. Bars represent means ± SE. The red dashed line in **(A, C)** marks the WT average, and the white dashed line in **(B, D)** indicates the height of WT plants. The statistical significance compared with WT was determined by one-way ANOVA followed by Tukey’s test; ***P < 0.001. Scale bar = 20 cm.

To investigate the functional consequences of Class II *GA2ox* gene disruption, these variants were evaluated in the paddy field for a range of agronomic traits. In *osga2ox1*, the relative plant height (RPH) at maturity was significantly increased in the KO variant compared to the wild type (WT), while the KD variants displayed RPH values similar to WT (**Figures 1A, B**). Detailed trait analyses of the KO line confirmed a ~5.5% increase in plant height, but no differences in heading date (HD), panicle number (PN), grain number per panicle (GNP), seed setting rate (SSR), 1000-grain weight (TGW), or yield per plant (YPP) compared with WT (**Table 2**).

**Table 2:** Agronomic trait comparisons between *ga2oxs* KO mutants and the WT host.

Mutants/WT	RPH (%)	N	HD (DAI)	PN^A^	GNP^B^	SSR (%)^C^	TGW (g)^D^	YPP^E^
**WT**	**100.0 ± 4.1**	**60**	**87.9 ± 2.0**	**7.1 ± 1.0**	**138.0 ± 12.1**	**91.5 ± 2.6**	**24.7 ± 0.5**	**22.1 ± 3.9**
*ga2ox1*	105.5 ± 5.6***	26	89.6 ± 2.2	7.4 ± 1.7	125.9 ± 9.6	92.9 ± 1.8	24.6 ± 0.9	21.3 ± 3.9
*ga2ox2*	100.0 ± 5.2	43	88.9 ± 3.3	6.7 ± 0.9	127.9 ± 13.4	93.0 ± 3.7	24.6 ± 0.9	19.6 ± 4.3
*ga2ox3*	105.4 ± 3.3***	44	85.3 ± 3.3	6.1 ± 1.2	136.3 ± 20.9	92.1 ± 2.9	23.9 ± 1.0	18.3 ± 2.5**
*ga2ox4*	108.2 ± 3.8***	41	89.1 ± 3.8	7.1 ± 1.5	121.9 ± 15.7	90.9 ± 3.9	24.3 ± 0.9	19.1 ± 3.3*
*ga2ox7*	102.4 ± 3.9	30	NA	6.9 ± 0.8	134.7 ± 13.0	90.0 ± 3.5	24.1 ± 0.9	20.2 ± 2.7
*ga2ox8*	104.6 ± 5.8**	35	NA	6.8 ± 1.3	140.2 ± 18.9	88.8 ± 5.1	23.7 ± 0.8	20.1 ± 2.6
*ga2ox5*	104.6 ± 4.9**	20	85.6 ± 4.2	6.8 ± 1.4	120.1 ± 16.0*	86.5 ± 5.1*	25.0 ± 1.0	17.7 ± 4.9**
*ga2ox6*	114.4 ± 6.2***	29	83.1 ± 2.1**	6.2 ± 0.8	119.2 ± 16.3*	93.6 ± 1.1	26.9 ± 0.6***	18.6 ± 2.2**
*ga2ox9*	103.0 ± 3.5	31	84.8 ± 3.4	6.9 ± 0.8	127.7 ± 10.7	90.2 ± 4.2	24.6 ± 0.9	19.6 ± 3.5

RPH, relative plant height; N, number of the samples used for RPH statistical analysis; HD, heading date; DAI, days after imbibition; NA, not available. Yield-related traits: PN, panicle numbers; GNP, grain number per panicle; SSR, seed setting rate. TGW: 1000-grain weight. YPP, yield per plant. Grain yield E = (A x B x C/100 x D)/1000. Agronomic traits other than RPH were evaluated with at least 3 replicates in a sample size of n≧4. The values are the means ± SD. The significant differences compare to WT using Dunnett’s test are indicated *p < 0.05, **p < 0.01, ***p < 0.001.

Bold values indicate the respective agronomic data of the wild type (WT).

By contrast, none of the *osga2ox2* KO lines showed detectable changes in plant height relative to WT (**Figures 1C, D**). An agronomic trait evaluation of the KO lines further confirmed that loss of *OsGA2ox2* alone did not affect HD, PN, GNP, SSR, TGW, or YPP (**Table 2**).

### 3.3 Phenotypic assessment of class II *OsGA2ox* genes using double-gene knockout mutants

To examine potential redundancy between Class II *OsGA2ox* genes, *osga2ox1/2* double-gene mutants were generated using a CRISPR/Cas9 construct targeting both *OsGA2ox1* and *OsGA2ox2* simultaneously. Three independent *osga2ox1/2* variant lines were obtained (**Table 3**). Lines “1.1” and “2.1” were homozygous double-gene knockout variants, with both genes carrying the “+1” frameshift allele, while line “7.1” carried a combination of the *osga2ox1* “+1” allele and a bi-allelic *osga2ox2* genotype (“-1/+1”).

**Table 3:** Collections of the multiple-gene KO mutants from variants with different allelic types and the number of samples used for statistical analysis.

Multiple-gene KO mutants	Lines/ variants	Allelic types on class II *GA2ox* genes				Sample sizes
		*2ox1*		*2ox2*		
2/1 Double	1.1	+1		+1		12
	2.1	+1		+1		12
	7.1	+1		-1/+1		11

Multiple-gene KO mutants	Lines/ variants	Allelic types on class I *GA2ox* genes				Sample sizes
		*2ox3*	*2ox4*	*2ox7*	*2ox8*	
3/4 Double	1.1	-2/+1	-29/-29			1
	1.2	-2/-2	-2/+1			1
	3.2	-1/-1	-2/-2			3
3/8	6.3	-2/-2			-1/-1	1
	6.4	-2/+1			-1/+1	1
	10.1	+1/+1			+4/+4	1
	10.2	+1/+1			-35/-35	2
4/7	5.1		-1/-1	+1/+1		1
	5.2		+1/+1	-30/-30		2
	6.1		-1/-1	+1/+1		9
7/8	2.1			-1/-1	+1/+1	6
	2.2			-27/-1	+1/+1	2
	12.1			-1/-1	+1/+1	9
3/4/7 Triple	2.2	+1/+1	-2/-2	+1/+1		1
	2.3	+1/+1	-2/-2	-1/-1		3
	4.1	-4/-4	-2/-2	-5/-5		8
3/4/8	1.1	+1/+1	-1/+1		-1/-1	1
	6.3	-2/+1	+2/+2		+1/+1	1
	7.1	-1/+1	-2/-2		-7/-7	2
	8.1	-1/-1	-2/-2		+1/+1	13
3/7/8	3.1	-22/-22		+1/+1	-7/-7	4
	10.2	-13/-13		-2/-2	-3/-3	1
	13.1	+1/+1		+1/+1	-3/-3	10
	27.1	-13/-13		-5/+17	-3/+2	1
4/7/8	11.1		+1/+1	-1/+1	-14/-14	15
	16.1		+20/+20	-8/-8	-1/-1	10
	17.1		+1/+1	-1/-1	+1/+1	15
	20.1		-1/-1	+1/+1	+1/+1	2
3/4/7/8 Quadruple	2.1	+1/+1	-2/-2	+1/+1	+1/+7	4
	2.2	+1/+1	-2/-2	-5/-1	-13/-13	3
	2.3	+1/+1	-2/-2	-1/+2	+1/+1	1
	5.1	+1/+1	-2/-2	+1/+1	+1/+1	6
	5.2	+1/+1	-2/-2	-5/+1	+1/+1	2

**Figure 2:**
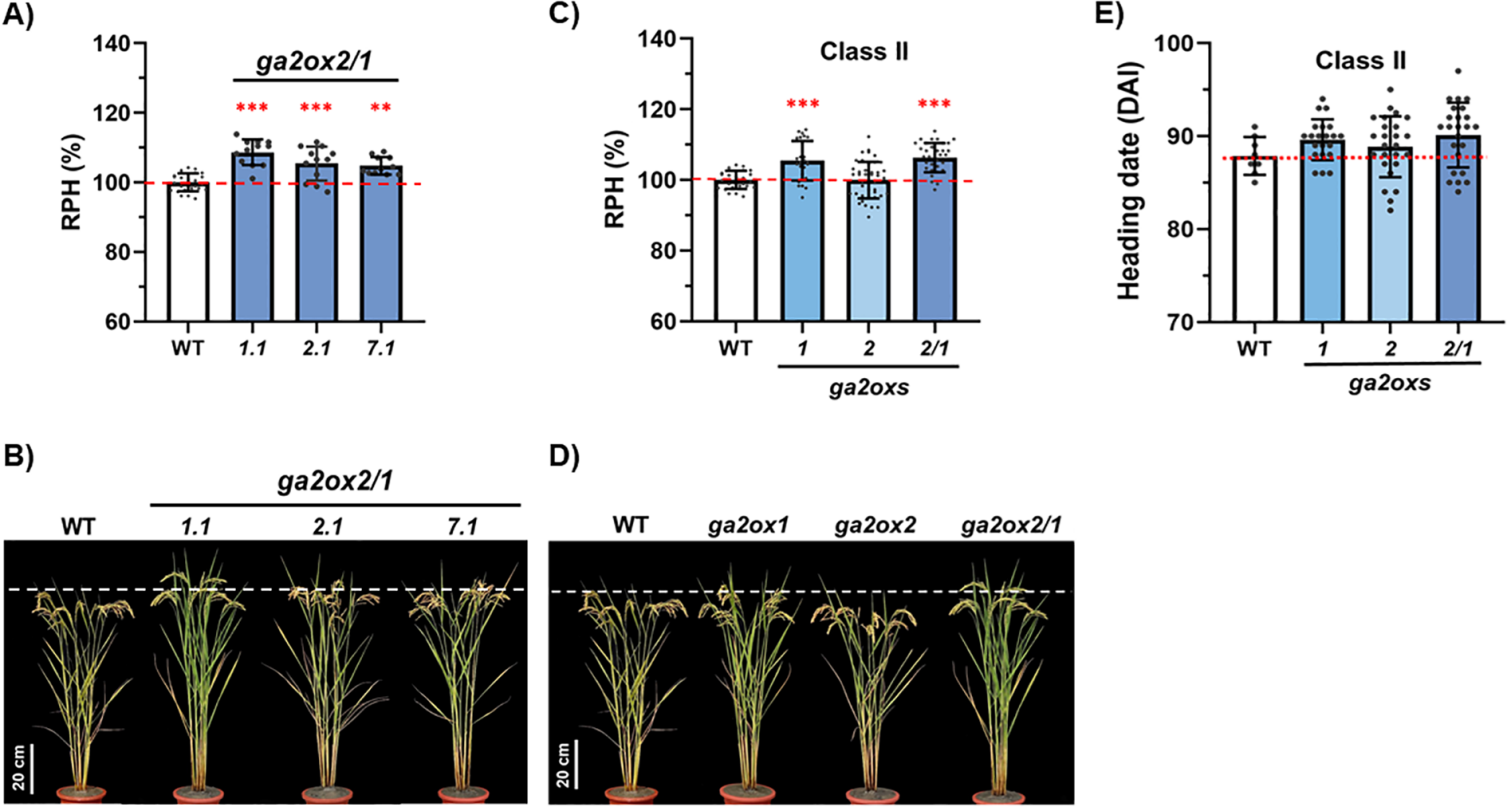
Effects of combined *osga2ox1* and *osga2ox2* knockouts on plant height and heading date. **(A, B)** Relative plant heights (RPH, %) and representative plants of the wild type (WT) and independent *osga2ox2/1* double mutants. **(C, D)** Comparison of RPHs in class II *osga2ox* single (*osga2ox1*, *osga2ox2*) and double (*osga2ox2/1*) mutants. **(E)** Comparison of heading dates in class II *osga2ox* single (*osga2ox1*, *osga2ox2*) and double (*osga2ox2/1*) mutants. DAI: days after imbibition. Bars represent means ± SE. The red dashed line marks the WT average, and the white dashed line indicates the height of WT plants. The statistical significance compared with WT was determined by one-way ANOVA followed by Tukey’s test; **P < 0.01, ***P < 0.001. Scale bar = 20 cm.

Our field evaluation of the double-gene mutants demonstrated that the relative plant height (RPH) at maturity was significantly increased in all *osga2ox1/2* lines compared with WT (**Figures 2A, B**). To further clarify the contributions of double- versus single-gene knockouts, the agronomic traits of the *osga2ox1/2* KO lines were compared with those of the *osga2ox1* and *osga2ox2* single-gene KO mutants. The RPH of the double-gene mutant was comparable to that of the *osga2ox1* single-gene KO mutant (**Figures 2C, D**), indicating *OsGA2ox1* as the major contributor to plant height regulation within Class II.

In contrast to plant height, WT, *osga2ox1*, *osga2ox2*, and *osga2ox1/2* flowered at similar times; although the double mutant showed a slight delay, the difference was not significant (**Figure 2E**; **Table 4**). In addition, the double-gene mutant displayed phenotypic differences not observed in the single-gene knockouts. For example, the grain number per panicle (GNP) was significantly reduced in the double-gene mutant (121 compared with 138 in WT; **Table 4**).

**Table 4:** Agronomic trait comparisons between multiple-*ga2ox-*gene KO mutants and the WT host.

Mutants/WT	RPH (%)	N	HD (DAI)	PN^A^	GNP^B^	SSR (%)^C^	TGW (g)^D^	YPP^E^
**WT**	**100.0 ± 4.1**	**60**	**87.9 ± 2.0**	**7.1 ± 1.0**	**138.0 ± 12.1**	**91.5 ± 2.6**	**24.7 ± 0.5**	**22.1 ± 3.9**
*ga2ox2/1*	106.3 ± 4.1***	35	90.1 ± 3.5	7.2 ± 1.0	121.4 ± 12.3**	89.3 ± 2.8	24.8 ± 1.1	19.4 ± 3.7
*ga2ox3/4*	120.6 ± 7.7***	5	87.6 ± 3.8	6.8 ± 0.8	158.2 ± 14.3	90.3 ± 3.5	24.1 ± 1.6	23.3 ± 2.8
*ga2ox3/8*	111.6 ± 7.4***	5	89.0 ± 2.2	6.8 ± 1.5	131.8 ± 25.5	92.3 ± 3.2	22.8 ± 1.2	18.9 ± 2.6
*ga2ox4/7*	99.3 ± 5.3	12	86.7 ± 3.5	7.2 ± 1.0	150.8 ± 31.4	86.7 ± 8.3	22.4 ± 1.4**	21.1 ± 6.3
*ga2ox7/8*	95.7 ± 6.4*	17	92.5 ± 2.1	7.5 ± 1.4	150.9 ± 18.8	81.8 ± 6.0**	21.7 ± 0.7***	20.1 ± 4.2
*ga2ox3/4/7*	111.9 ± 6.7***	12	86.6 ± 3.8	7.0 ± 1.4	145.5 ± 17.1	90.0 ± 2.3	23.0 ± 0.9	21.1 ± 3.3
*ga2ox3/4/8*	116.6 ± 7.9***	17	89.2 ± 5.5	7.5 ± 2.3	130.2 ± 18.2	90.0 ± 4.2	24.1 ± 1.6	21.2 ± 3.7
*ga2ox3/7/8*	101.4 ± 5.5	16	84.3 ± 2.9	7.8 ± 1.3	145.7 ± 19.7	86.2 ± 3.4	21.1 ± 1.0***	20.7 ± 4.3
*ga2ox4/7/8*	103.4 ± 5.1*	42	91.5 ± 4.4	6.5 ± 1.7	162.9 ± 25.9	90.1 ± 0.4	22.3 ± 1.3**	21.3 ± 6.0
*ga2ox3/4/7/8*	122.3 ± 4.8***	16	85.0 ± 2.4	7.0 ± 1.3	166.9 ± 38.0	83.6 ± 4.5*	21.7 ± 0.5***	21.2 ± 3.0

RPH, relative plant height; N, number of the samples used for RPH statistical analysis; HD, heading date; DAI, days after imbibition; NA, not available. Yield-related traits: PN, panicle numbers; GNP, grain number per panicle; SSR, seed setting rate; TGW, 1000-grain weight; YPP, yield per plant. Grain yield E = (A x B x C/100 x D)/1000. Agronomic traits other than RPH were evaluated with at least 3 replicates in a sample size of n≧4. The values are the means ± SD. The significant differences compare to WT using Dunnett’s test are indicated *p < 0.05, **p < 0.01, ***p < 0.001.

Bold values indicate the respective agronomic data of the wild type (WT).

### 3.4 Phenotypic assessment of class I *OsGA2ox* genes using single-gene knockout mutants

To investigate the roles of class I *OsGA2ox* genes, a total of 18 CRISPR/Cas9 variant lines were generated for four functional members (**Table 1**). For *OsGA2ox3*, five variant lines were obtained, including two homozygous knockout (KO) lines (“+1” and “-2” variants), two bi-allelic KO lines (“-2/+1” and “-13/+1” variants), and one in-frame deletion mutant (“-3” variant) (**Supplementary Figure S3-3**). For *OsGA2ox4*, five homozygous KO lines were generated, comprising two “+1” variants, two “+2” variants, and one “-38” variant (**Supplementary Figure S3-4**). For *OsGA2ox7*, four homozygous KO lines were identified, corresponding to the “+1,” “+43,” “-2,” and “-5” variants (**Supplementary Figure S3-7**). For *OsGA2ox8*, three homozygous KO lines (“+1,” “-19,” and “-41” variants) along with one in-frame deletion mutant (“-33” variant) were obtained (**Supplementary Figure S3-8**).

**Figure 3:**
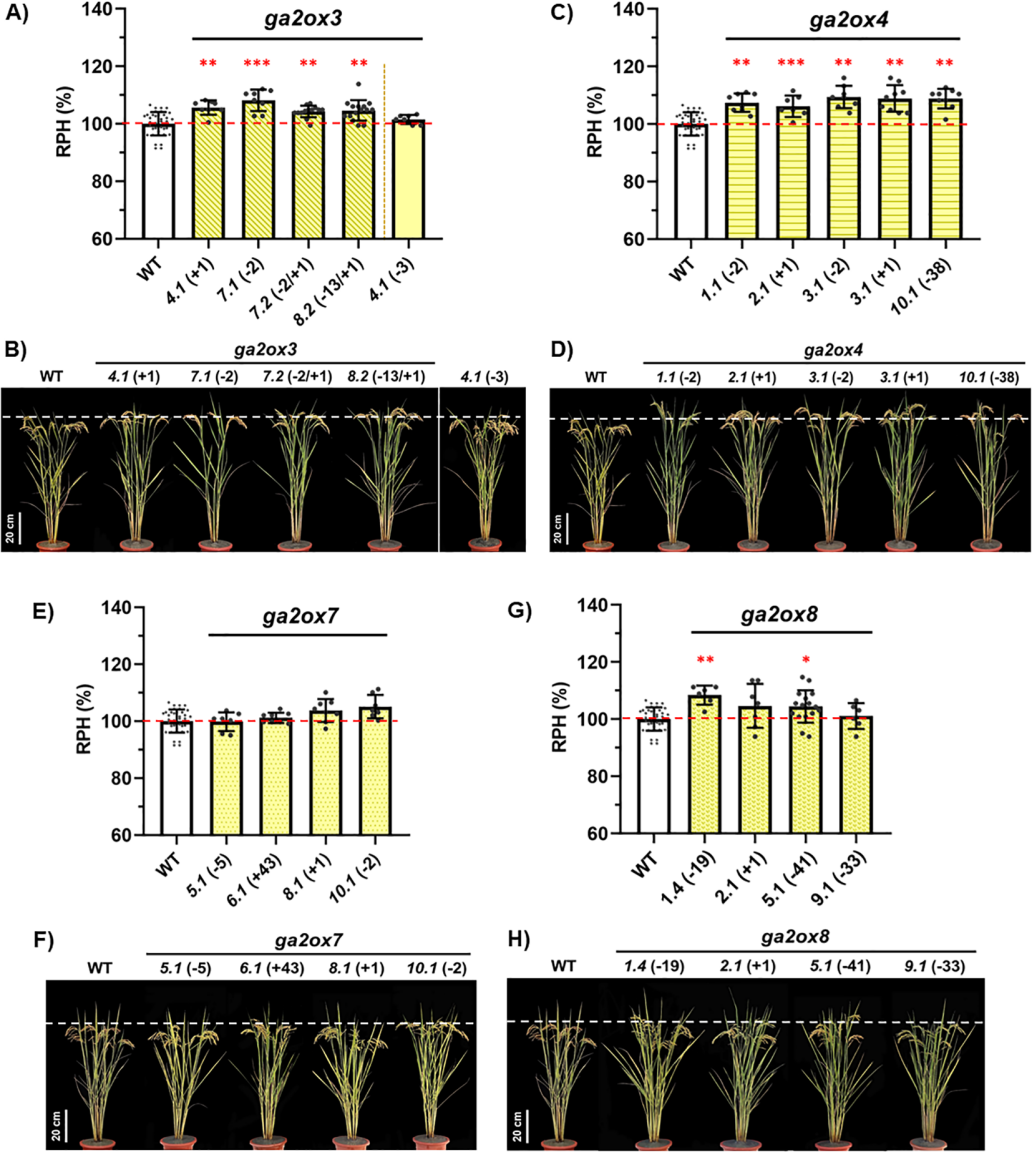
Impact of Class I *osga2ox* mutants on plant height. **(A, B)** Analysis of *osga2ox3* mutants. **(A)** Relative plant heights (RPH, %) of the wild type (WT) and independent *osga2ox3* mutant lines. **(B)** Representative plants of WT and *osga2ox3* mutants. The genotypes and line identifiers are shown. **(C, D)** Analysis of *osga2ox4* mutants. **(C)** RPHs of WT and independent *osga2ox4* mutant lines. **(D)** Representative plants of WT and *osga2ox4* mutants. **(E, F)** Analysis of *osga2ox7* mutants. **(E)** RPHs of WT and independent *osga2ox7* mutant lines. **(F)** Representative plants of WT and *osga2ox7* mutants. **(G, H)** Analysis of *osga2ox8* mutants. **(G)** RPHs of WT and independent *osga2ox8* mutant lines. **(H)** Representative plants of WT and *osga2ox8* mutants. Bars represent means ± SE. The red dashed line marks the WT average, and the white dashed line indicates the height of WT plants. The statistical significance compared with WT was determined by one-way ANOVA followed by Tukey’s test; *P < 0.05, **P < 0.01, ***P < 0.001. Scale bar = 20 cm.

Relative plant heights (RPHs) were compared across the mutant lines within each gene group. Four *osga2ox3* KO lines showed significantly increased RPHs compared with WT, while the “-3” in-frame deletion mutant did not differ from WT (**Figures 3A, B**). All *osga2ox4* KO lines exhibited a higher RPH relative to WT (**Figures 3C, D**). In contrast, RPH was unchanged in *osga2ox7* KO mutants compared with WT (**Figures 3E, F**). For *osga2ox8*, two KO lines (“–19” and “–41”) showed significantly increased RPH compared with WT, whereas the “+1” variant exhibited a similar trend but did not reach statistical significance (**Figures 3G, H**). These findings demonstrated that *OsGA2ox3*, *OsGA2ox4*, and *OsGA2ox8* knockouts significantly influenced RPH, whereas the *OsGA2ox7* knockout had no measurable effect. Apart from differences in RPH, the analysis of yield-related agronomic trait revealed differences in yield per plant (YPP) for *osga2ox3* and *osga2ox4*, whereas all other measured agronomic traits showed no significant differences between the mutants and WT (**Table 2**).

### 3.5 Phenotypic assessment of class I *OsGA2ox* genes using multiple-gene knockout mutants

To investigate functional redundancy among class I *OsGA2ox* genes, we generated a series of multiple-gene knockout (KO) mutants and evaluated their collective effects on plant growth and agronomic traits. Among the 11 possible combinations of double, triple, and quadruple KO mutants, nine combinations were successfully obtained, exception for the double mutants *osga2ox3/7* and *osga2ox4/8* (**Table 3**). For each multiple-gene KO genotype, at least two independent variants were established, with sample sizes ranging from 5 to 42. All variants of the same genotype were pooled for statistical analysis of plant growth effects.

**Figure 4:**
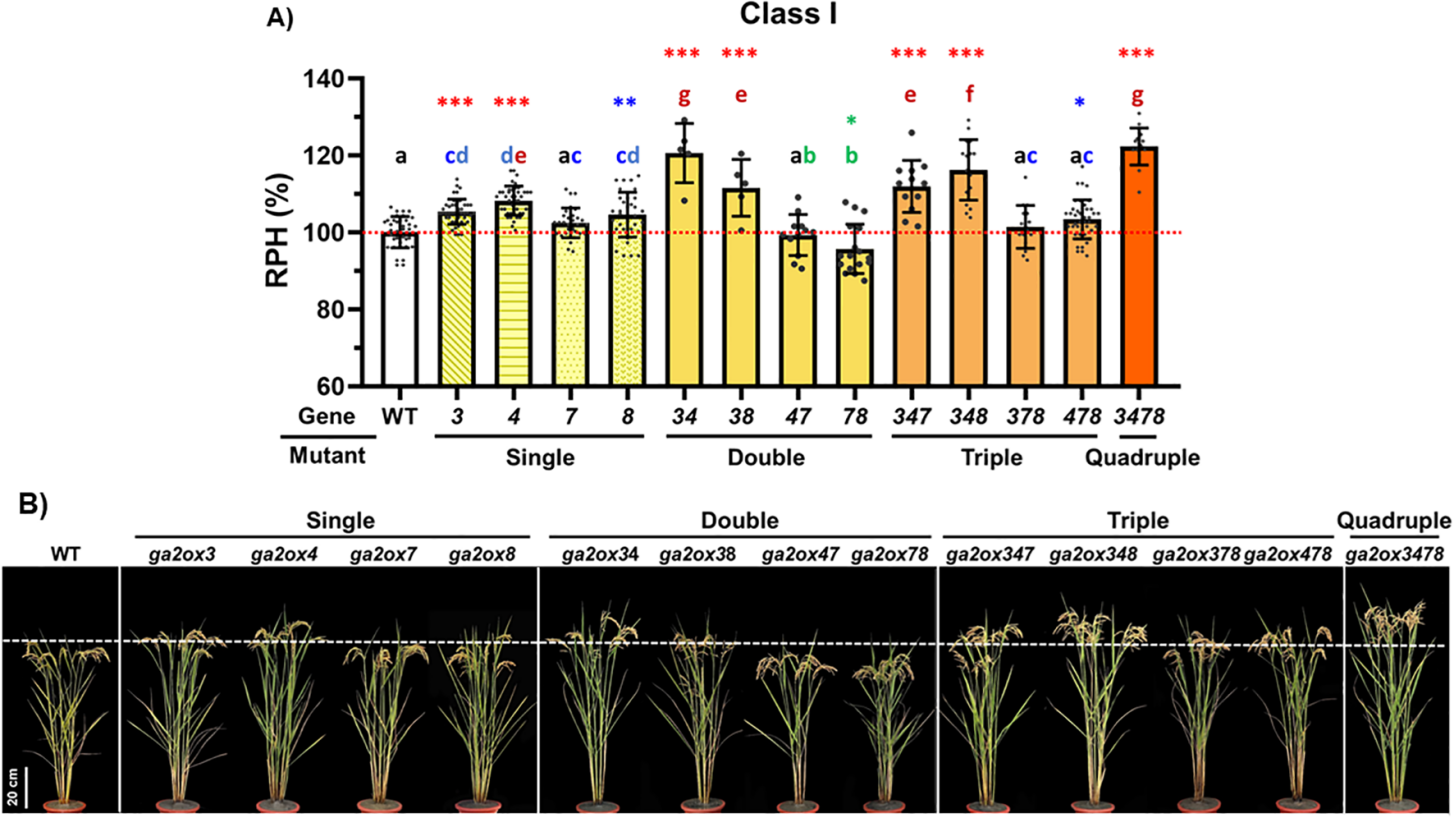
Cumulative effects of Class I *osga2ox* mutations on rice plant height. **(A)** Relative plant heights (RPH, %) of the wild type (WT) and Class I *osga2ox* mutants. Bars represent means ± SE (n = 5–42 plants per genotype). Mutants are grouped into single (*osga2ox3, osga2ox4, osga2ox7, osga2ox8*), double (*osga2ox34, osga2ox38, osga2ox47, osga2ox78*), triple (*osga2ox347, osga2ox348, osga2ox378*), and quadruple (*osga2ox3478*) mutant combinations. The red dashed line marks the WT average. The letters above bars denote statistically significant differences among genotypes (*P* < 0.05) based on multiple comparison testing. The asterisks indicate significance relative to WT (*P < 0.05, **P < 0.01, ***P < 0.001). **(B)** Representative plants of WT and Class I *osga2ox* mutants.

Relative plant heights (RPHs) were compared between multiple-gene KO mutants, single-gene KO mutants, and WT at the mature stage (**Figure 4**). Among the double-gene mutants, RPH was increased in *osga2ox3/4* and *osga2ox3/8*, but decreased in *osga2ox4/7* and *osga2ox7/8*. Similar patterns were observed in triple-gene KO mutants: the RPH of *osga2ox3/4/8* was comparable to that of *osga2ox3/4* but higher than that of *osga2ox3/8*, whereas the RPHs of *osga2ox3/7/8* and *osga2ox4/7/8* were greater than those of *osga2ox7/8* and *osga2ox4/7*. In contrast, the RPH of *osga2ox3/4/7* was lower than that of *osga2ox3/4*. Notably, the quadruple-gene KO mutants exhibited the greatest increase in RPH compared with all other multiple-gene KO mutants.

Our agronomic trait analysis of multiple-gene KO mutants relative to WT (**Table 4**) revealed a reduction in thousand-grain weight (TGW) in *osga2ox4/7*, *osga2ox7/8*, *osga2ox3/7/8*, *osga2ox4/7/8* and *osga2ox3/4/7/8*. Additionally, a decrease in seed-setting rate (SSR) was observed specifically in *osga2ox7/8* and *osga2ox3/4/7/8*.

Together, these findings suggest that class I *OsGA2ox* genes act redundantly to regulate plant height, but their combined disruption can compromise yield-related traits such as TGW and SSR.

### 3.6 Phenotypic assessment of class III *OsGA2ox* genes using single-gene knockout mutants

**Figure 5:**
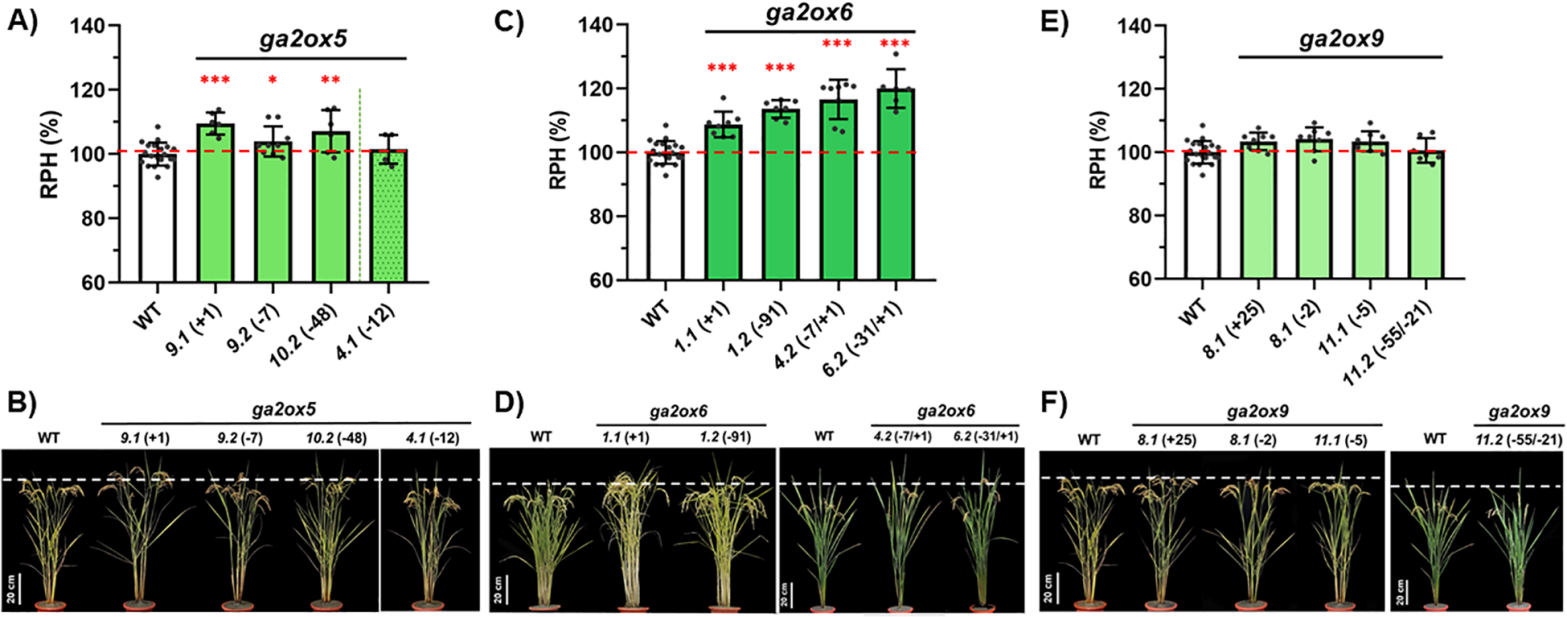
Plant height phenotypes of Class III *osga2ox* mutants. **(A, B)** Analysis of *osga2ox5* mutants. **(A)** Relative plant heights (RPH, %) of the wild type (WT) and independent *osga2ox5* mutant lines. **(B)** Representative plants of WT and *osga2ox5* mutants. The genotypes and line identifiers are shown above. **(C, D)** Analysis of *osga2ox6* mutants. **(C)** RPHs of WT and independent *osga2ox6* mutant lines. **(D)** Representative plants of WT and *osga2ox6* mutants. **(E, F)** Analysis of *osga2ox9* mutants. **(E)** RPHs of WT and independent *osga2ox9* mutant lines. **(F)** Representative plants of WT and *osga2ox9* mutants. Bars represent means ± SE. The red dashed line marks the WT average, and the white dashed line indicates the height of WT plants. The statistical significance compared with WT was determined by one-way ANOVA followed by Tukey’s test; *P < 0.05, **P < 0.01, ***P < 0.001. Scale bar = 20 cm.

To elucidate the functional roles of class III *OsGA2ox* genes, we generated a set of single-gene knockout (KO) mutants and systematically evaluated their effects on rice growth and development. In total, 12 variant lines were established (**Table 1**). For *OsGA2ox5*, two homozygous KO lines (the “+1” and “–7” variants) and two homozygous in-frame deletion lines (the “–12” and “–48” variants) were obtained (**Supplementary Figure S3-5**). Compared to WT, RPH was increased in the “+1” and “–7” KO variants and in the “–48” in-frame deletion variant, but not increased in the “–12” variant (**Figures 5A, B**). For *OsGA2ox6*, two homozygous KO lines (the “+1” and “–91” variants) and two bi-allelic KO lines (the “–7/+1” and “–31/+1” variants) were produced (**Supplementary Figure S3-6**). All variant lines, regardless of homozygous or bi-allelic, exhibited an increased RPH compared to WT (**Figures 5C, D**). For *OsGA2ox9*, three homozygous KO lines (the “+25,” “–2,” and “–5” variants) and one bi-allelic line (the “–55/–21” variant) were identified (**Supplementary Figure S3-9**). In contrast to *OsGA2ox5* and *OsGA2ox6*, all *osga2ox9* variants showed RPHs similar to WT (**Figures 5E, F**).

All KO lines of class III genes were used for further agronomic evaluation. In *osga2ox5*, the grain number per panicle (GNP) and the seed-setting rate (SSR) were reduced (from 138.0 and 91.5% in WT to 120.1 and 86.5%, respectively), leading to a decrease in yield per plant (YPP) from 22.1 to 17.7 (**Table 2**). In *osga2ox6*, the reductions in GNP (by 18.8, from 138.0 in WT to 119.2) and YPP (by 3.5, from 22.1 to 18.6) were also observed, although SSR was unchanged. Notably, the *osga2ox6* mutants exhibited earlier heading (83.1 vs. 87.9 days after imbibition in WT) and increased thousand-grain weight (TGW; 26.9 vs. 24.7) (**Table 2**). In contrast, no significant differences in agronomic traits were detected in the *osga2ox9* mutants compared with WT (**Table 2**).

**Figure 6:**
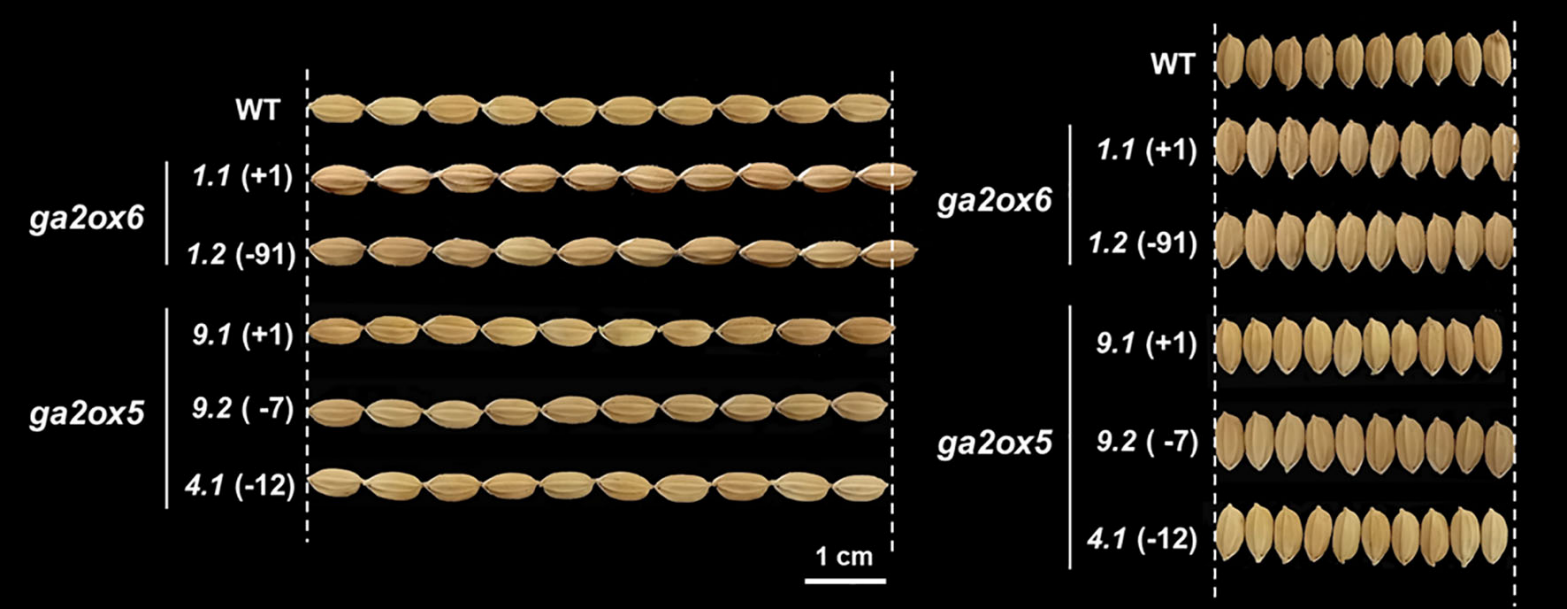
Seed size comparison of WT, *osga2ox5*, and *osga2ox6* mutants. Representative seed images of the wild type (WT) and independent *osga2ox5* and *osga2ox6* variant lines. The genotypes and the line identifiers are indicated on the left. Seeds are shown in horizontal (left panel) and vertical (right panel) views for each genotype. Scale bar = 1 cm.

To further validate the early heading phenotype, all *osga2ox6* variants were re-examined in a different growing season. The “+1/+1” variant headed at 94.9 ± 1.9 days after imbibition (DAI), the “–91/–91” variant at 94.2 ± 1.6 DAI, the “–7/–7” variant at 93.2 ± 1.6 DAI, and the “–31/+1” variant at 95.5 ± 2.1 DAI, all significantly earlier than the wild type (97.1 ± 2.1 DAI) (**Table 5**). Thus, HD was advanced by 1.6–3.9 days in *osga2ox6* mutants compared with WT. Seed size was further evaluated in the “+1/+1” and “–91/–91” variants. While the seed width was unchanged, the seed length increased by 0.16-0.36 mm relative to WT (**Figure 6**; **Table 5**). These findings demonstrate that *osga2ox6* not only promotes plant height but also accelerates heading and enhances TGW, likely through the production of longer grains.

**Table 5:** Heading date and seed characteristics of *ga2ox6* KO variants and the WT host.

WT/Variants		HD (DAI)	N	AS (mm^2^)	PL (mm)	Length (mm)	Width (mm)	L/W
**WT**		**97.1 ± 2.1**	**17**	**20.6 ± 1.8**	**19.5 ± 1.1**	**7.51 ± 0.49**	**3.82 ± 0.20**	**1.97 ± 0.15**
*ga2ox6*	*1.1* (+1/+1)	94.9 ± 1.9**	16	21.3 ± 2.1**	19.8 ± 1.1**	7.67 ± 0.44**	3.79 ± 0.24	2.03 ± 0.15***
	*1.2* (-91/-91)	94.2 ± 1.6***	18	22.1 ± 1.7***	20.1 ± 0.9***	7.87 ± 0.37***	3.81 ± 0.17	2.07 ± 0.13***
	*4.2 (-7/-7)*	93.2 ± 1.6***	19	ND	ND	ND	ND	ND
	*6.2 (-31/+1)*	95.5 ± 2.1*	19	ND	ND	ND	ND	ND

HD, Heading date; N, number of sample used for heading date analysis; AS, Area size; PL, Perimeter length; L/W, Length-to-width ratio; ND, Not determined; DAI, Days after imbibition. The values are the means ± SD (n≧150). The significant differences to WT using Dunnett’s test are indicated as *p < 0.05, **p < 0.01, ***p < 0.001.

Bold values indicate the respective agronomic data of the wild type (WT).

## 4 Discussion

The *GA2ox* family is the largest one among GA biosynthetic and catabolic genes in rice. While the roles of GA biosynthetic genes (e.g., *CPS*, *KS*, *KO*, *KAO*, *GA20ox*, and *GA3ox*) have been well-documented through studies of natural and *Tos17* insertion mutants (Sakamoto et al., 2004), the characterization of *GA2ox* genes has relied mainly on transgenic approaches (Lo et al., 2008; Hsieh et al., 2021). This is partly due to the lack of natural knockout (KO) mutants for *OsGA2ox* genes, which suggests prevalent functional redundancy within this gene family. Recent advances in gene editing have enabled the generation of *osga2ox* mutants using the CRISPR/Cas9 system to investigate their roles in rice growth and development (Chen et al., 2019; Takehara et al., 2020; Jin et al., 2023; Xing et al., 2023). However, differences in genetic backgrounds and growth conditions across these studies complicate direct comparisons of phenotypic outcomes. Moreover, because previous studies centered primarily on single-gene knockouts, they could not address how *GA2ox* paralogs interact, compensate for one another, or differ in their relative contributions, which are important factors for understanding family-wide redundancy and identifying dominant regulators within the *GA2ox* family.

To address these gaps, we generated a comprehensive set of 36 *osga2ox* mutant lines in a uniform genetic background: the rice cultivar *Oryza sativa* Tainung 67. We created 1–5 independent KO alleles for each *OsGA2ox* gene and, importantly, produced various combinations of KO variants, including class II double mutants and class I multiple mutants. This uniform genetic background allows for a direct and reliable comparison of the phenotypic effects of different *OsGA2ox* knockouts. By comparing single and multiple KO mutants from a gene class, we can comprehensively assess the dominant or minor contributions of specific genes within each class. Our use of multiple allelic variants and tracking of phenotypes across generations ensures that the observed changes are due to the loss of *OsGA2ox* function and not CRISPR/Cas9 off-target effects.

### 4.1 Functional roles of class II *GA2ox* genes in rice

Class II genes include *OsGA2ox1* and *OsGA2ox2*. *OsGA2ox1*, which was the first functional *GA2ox* gene identified in rice (Sakamoto et al., 2001), showed stronger GA-deactivation activity and so was considered the dominant paralog (Hsieh et al., 2021). *OsGA2ox2*, once thought to be a pseudogene (Sakai et al., 2003; Han and Zhu, 2011), was later found to be functional (Hsieh et al., 2021). Expression profiling indicates that *OsGA2ox1* is predominantly expressed in reproductive tissues, such as flowers, panicles, and anthers, whereas *OsGA2ox2* shows extremely low or barely detectable expression across the analyzed tissues (Hsieh et al., 2021). *OsGA2ox1* and *OsGA2ox2* did not show clear responsiveness to GA treatment in the available datasets (**Supplementary Figure S5**). However, the relative contributions of these two paralogs to rice growth was unclear.

#### 4.1.1 *OsGA2ox1* dominates plant height regulation

In the present study, *osga2ox1* knockout mutants exhibited a clear increase in relative plant height (RPH), whereas *osga2ox2* mutants were indistinguishable from WT (**Figure 1**). In rice, plant height is primarily determined by the elongation of internodes in the culm, with cell division occurring in the intercalary meristem, located approximately 2–4 mm above each node (Nagai et al., 2025). Although the overall expression of *OsGA2ox1* in stems is low (Hsieh et al., 2021), it has been detected in the stem node and intercalary meristem (Cao et al., 2024), suggesting that *OsGA2ox1* plays an important role in the regulation of plant height.

This interpretation is consistent with previous findings, which showed that loss-of-function of *OsGA2ox1* results in increased plant height in Nipponbare (Cao et al., 2024), indicating that its role in plant height regulation is conserved across Japonica cultivars. Furthermore, the *osga2ox1/2* double mutant exhibited a further, though modest, increase in RPH by 106.3% (**Table 4**), compared with 105.5% in *osga2ox1* (**Table 2**), suggesting that *OsGA2ox2* contributes a minor additive effect. This partial redundancy aligns with the concept of genetic buffering, as shown in tomato GID1 receptor mutants where overlapping paralogs stabilized GA signaling and maintained growth under variable environments (Illouz-Eliaz et al., 2019). Similarly, the small but detectable contribution of *OsGA2ox2* implies functional redundancy with *OsGA2ox1*, potentially providing a buffering mechanism to fine-tune GA homeostasis and maintain stable control of culm elongation under varying conditions. Collectively, these results demonstrate that *OsGA2ox1* is the principal regulator of plant height among class II genes, whereas *OsGA2ox2* plays only a subsidiary role.

#### 4.1.2 Loss of *OsGA2ox1/2* does not alter heading date in TNG67

Because *OsGA2ox1* is strongly expressed at the shoot apex, it has been hypothesized to regulate phase transition (Sakamoto et al., 2001). In maize, the ortholog *ZmGA2ox1* is repressed by *KN1* to delay flowering (Bolduc and Hake, 2009). In addition, GA is well established as promoters of flowering (Reeves and Coupland, 2001; Eriksson et al., 2006), acting at the shoot apical meristem (SAM) to induce expression of floral integrator genes (Bao et al., 2020). Notably, *OsGA2ox1* is expressed in a ring-shaped domain beneath the apical dome (Sakamoto et al., 2001), where it inactivates bioactive GAs, and its expression decreases markedly upon floral induction, suggesting that the SAM may become more accessible to GAs during reproductive development. Based on this expression pattern, we anticipated that *osga2ox1* mutants might exhibit earlier heading. Contrary to this expectation, heading date did not differ between *osga2ox1* and the wild type (WT) or the *osga2ox1/2* double mutant (**Figure 2E**, **Table 2**, **4**).

However, a previous study reported that knockout of *OsGA2ox1* accelerated flowering in Nipponbare (Cao et al., 2024), which is inconsistent with our findings. One possible explanation for this discrepancy is the difference in genetic background. Unlike the photoperiod-sensitive Nipponbare (Matsubara et al., 2008), TNG67 is a photoperiod-insensitive cultivar with defective *Hd1* and *Ehd1* alleles, similar to those found in TN65 (Doi et al., 2004; Wang et al., 2013). Notably, GA and photoperiod pathways have been shown to act synergistically to regulate flowering under long-day conditions in Arabidopsis (Wang et al., 2016). This suggests that the impact of GA metabolism on flowering may be strongly influenced by the status of the photoperiod pathway. In cultivars such as TNG67, where photoperiod regulation is compromised, the contribution of class II *GA2ox* genes to heading may therefore be attenuated. Taken together, these findings suggest that class II *GA2ox* genes may be dispensable for heading regulation in photoperiod-insensitive cultivars such as TNG67.

#### 4.1.3 Loss of *OsGA2ox1/2* does not compromise seed setting but reduces grain number per panicle in TNG67

*OsGA2ox1* is predominantly expressed in anthers and panicles (Hsieh et al., 2021), suggesting potential roles in reproductive development. However, our previous analysis showed that loss of *OsGA2ox1* did not alter pollen morphology or fertility (Hsieh et al., 2021). Consistent with this, we found that seed setting rate (SSR) in both *osga2ox1* and *osga2ox2* single mutants, as well as in the *osga2ox1/2* double mutant, was comparable to that of the wild type (**Tables 2**, **4**), indicating that Class II *GA2ox* genes do not significantly affect fertilization or seed formation. Although SSR remained unaffected, Class II genes influenced another major reproductive trait, i.e., the grain number per panicle (GNP).

GA is known to regulate inflorescence meristem activity and branch elongation, and perturbations in GA biosynthesis or signaling often affect panicle architecture (Deveshwar et al., 2020; Chun et al., 2022). In our study, single mutations in *OsGA2ox1* or *OsGA2ox2* caused only a mild downward trend in GNP that did not reach statistical significance (**Table 2**). In contrast, the *osga2ox1/2* double mutant exhibited a significant reduction in GNP compared with the wild type (121.4 vs. 138.0; **Table 4**), demonstrating that the two Class II paralogs function redundantly to maintain normal panicle productivity.

Recent work also supports a role for *OsGA2ox1* in panicle development. Loss of the upstream repressor *OsBLH4* was shown to decrease GA_1_ levels in young panicles and to affect the expression of panicle-related genes, linking *OsGA2ox1* to GA homeostasis during panicle development (Cao et al., 2024). Elevated *OsGA2ox1* expression suppresses *GNP1/OsGA20ox1* and *IPA1* (Lu et al., 2013; Wu et al., 2016; Cao et al., 2024), suggesting that increased GA catabolism may reduce cytokinin signaling and thereby lower GNP. Interestingly, *osga2ox1* in Nipponbare increased GNP (Cao et al., 2024), contrasting with our findings in TNG67, where the single mutant showed no significant change and reduced GNP was observed only in the double mutant. These contrasting outcomes likely reflect genetic background effects, as cultivar-specific regulatory networks may influence how loss of *OsGA2ox1* impacts meristem activity and branch formation (Gaarslev et al., 2021). Taken together, these results indicate that *OsGA2ox1* and *OsGA2ox2* do not impair seed setting but act redundantly to maintain the grain number per panicle in a genotype-dependent manner.

### 4.2 Functional roles of class I *GA2ox* genes in rice

Class I is the largest group of *GA2ox* genes in rice and is divided into two clades (Hsieh et al., 2021). Clade A members (*OsGA2ox3*, *OsGA2ox4*, and *OsGA2ox8*) exhibit strong GA-deactivation activity, whereas the clade B gene *OsGA2ox7* shows only weak activity (Hsieh et al., 2021; Jin et al., 2023). Among them, *OsGA2ox3* has been proposed as the dominant paralog due to its ubiquitous expression and minimal sequence variation. In contrast, *OsGA2ox4*, *OsGA2ox8*, and *OsGA2ox7* display relatively low expression levels across most tissues (Hsieh et al., 2021). GA responsiveness was primarily observed in Class I genes, with clear hierarchical differences (**Supplementary Figure S5**). *OsGA2ox3* responded rapidly within 1 hour and peaked at 12 hours, whereas *OsGA2ox4* and *OsGA2ox7* showed delayed induction at 12 hours. In contrast, *OsGA2ox8* exhibited a gradual increase from 3 to 12 hours. However, the relative developmental contributions of the individual Class I paralogs remain not fully elucidated.

#### 4.2.1 Paralog-specific contributions of clade A *GA2ox* genes to rice plant height

To clarify the relative contributions of individual class I *GA2ox* paralogs to plant height regulation, we examined the phenotypic consequences of 17 KO mutants in class I genes. Although *osga2ox3*, *osga2ox4*, and *osga2ox8* exhibited increased RPH (**Figures 3A, C, E**), the plant height increase differed among paralogs, with RPH increases of 5.4%, 8.2%, and 4.6%, respectively (**Table 2**), indicating differential contributions to GA catabolism and stem elongation control. Both *OsGA2ox4* and *OsGA2ox8* are considered regulatory hypofunctionalized duplicates derived from *OsGA2ox3* (Hsieh et al., 2021), and the low expression of *OsGA2ox8* across tissues, together with its minor effect on plant height (**Table 2**), supports the hypothesis that *OsGA2ox8* represents a hypofunctionalized duplicate of *OsGA2ox3*. Surprisingly, the knockout of *OsGA2ox4* resulted in a greater RPH increase than that observed in *osga2ox3* (**Table 2**). This may reflect divergence in expression pattern and broader substrate specificity of *OsGA2ox4*. Whereas *OsGA2ox3* is expressed ubiquitously across tissues (Sakai et al., 2003; Hsieh et al., 2021), *OsGA2ox4* expression is primarily localized to the basal region of the internode (Liu et al., 2018), enabling efficient regulation of bioactive GA flow to the intercalary meristematic region. In addition, *OsGA2ox4* can catalyze GA_19_, GA_20_, and GA_1_, whereas *OsGA2ox3* targets only GA_20_ and GA_1_ (Sakai et al., 2003; Liu et al., 2018). Moreover, natural amino acid variants in *OsGA2ox4* have been associated with up to ~26% reduction in RPH (Liu et al., 2018), underscoring its strong role in plant height regulation.

#### 4.2.2 Dominant contributions of *OsGA2ox3* and *OsGA2ox4* to plant height regulation

The distinct effects of individual class I *GA2ox* paralogs on plant height prompted us to investigate whether these genes function independently or exhibit overlapping activity. Functional redundancy among gene family members can obscure the contribution of individual genes; therefore, elucidating their specific roles requires analysis of multiple knockout combinations. Indeed, similar approaches in the GID1 receptor and *GA20ox* gene families have demonstrated that loss of a single gene generally results in no or only mild phenotypic alteration, whereas simultaneous disruptions of multiple redundant paralogs lead to markedly enhanced phenotypes (Griffiths et al., 2006; Plackett et al., 2012; Illouz-Eliaz et al., 2019). These studies highlight synergistic interactions among family members and provide important insight into their cooperative roles in regulating GA-mediated growth.

Because single knockouts of *OsGA2ox3*, *OsGA2ox4*, and *OsGA2ox8* increased relative plant height to different extents, these clade A paralogs contribute unequally to plant height regulation, suggesting partial and unequal redundancy. However, whether these genes can genetically compensate for one another upon simultaneous disruption remained unclear. To address this question, we analyzed multiple-knockout combinations. Notably, the height increase in the *osga2ox3/4* and *osga2ox3/8* double mutants exceeded the expected additive outcome based on the corresponding single mutants. The *osga2ox3*, *osga2ox4*, and *osga2ox8* showed increases in RPH of 5.4%, 8.2%, and 4.6%, respectively (**Table 2**). Under additive effects, one expects increases of 13.6% for *osga2ox3/4* and 10.0% for *osga2ox3/8*. However, the observed RPH increases were 20.6% in *osga2ox3/4* and 11.6% in *osga2ox3/8* (**Table 4**), which exceeded the expected additive values by 7.0% and 1.6%, respectively. On the other hand, pairwise statistical comparisons among the multiple knockout lines showed that *osga2ox3*, *osga2ox4* and *osga2ox8* consistently increased plant height across multiple genetic backgrounds (**Supplementary Figures S4A, B, D**), indicating that these clade A paralogs generally contribute in an additive manner to RPH. However, these increases were not uniformly observed across mutant combinations. For example, the RPH of *osga2ox3/4/8* was not greater than that of the *osga2ox3/4* (**Table 4**; **Supplementary Figure S4D**), so that additive expectations are not consistently observed.

To evaluate these deviations from additivity, we performed factorial linear model analysis (**Supplementary Table S2**). A significant positive interaction between *OsGA2ox3* and *OsGA2ox4* was detected (P = 0.002), indicating a synergistic increase in plant height. In contrast, the *osga2ox3/8* combination did not show a significant interaction (P = 0.145), indicating that its effect is consistent with an additive model.

The synergistic effects between *OsGA2ox3* and *OsGA2ox4* found in double mutants suggest that their functional importance increases upon the loss of *OsGA2ox3*, reflecting a reduction in genetic buffering within the class I *GA2ox* family. This synergistic relationship among clade A genes may arise from a redundancy mechanism known as active compensation, in which the expression of one paralog is upregulated to counterbalance the functional loss of another (Iohannes and Jackson, 2023). Such active compensation has been reported in the *GA20ox* gene family of *Arabidopsis* (Rieu et al., 2008), suggesting that a similar compensatory mechanism may also operate among clade A *OsGA2ox* genes to maintain GA homeostasis.

Note that the increase in plant height in the *osga2ox3/4* double mutant was 9.0% greater than that in the *osga2ox3/8* double mutant (**Table 4**), indicating that loss of *OsGA2ox4* has a stronger impact on plant height than loss of *OsGA2ox8*. Moreover, introducing additional knockouts beyond *osga2ox3* and *osga2ox4* resulted in only marginal changes; RPH increased slightly, from 120.6% in the *osga2ox3/4* double mutant to 122.3% in the *osga2ox3/4/7/8* quadruple mutant (**Table 4**). This small increase in RPH suggests that the growth-promoting effect of class I *OsGA2ox* gene knockouts becomes nearly exhausted when both *OsGA2ox3* and *OsGA2ox4* are disrupted. In addition, pairwise statistical comparisons revealed that *OsGA2ox3* and *OsGA2ox4* exhibited stronger genetic interactions than *OsGA2ox8* (**Supplementary Figure S4D**). Together, these results indicate that the disruptions of *OsGA2ox3* and *OsGA2ox4* account for most of the growth-promoting effect observed in clade A knockouts, underscoring their roles as the dominant paralogs within the class I *GA2ox* genes.

#### 4.2.3 Clade B gene *OsGA2ox7* exhibits context-dependent effects

While clade A genes show additive or synergistic genetic interactions, the functional behavior of clade B genes appears markedly different. The clade B gene *OsGA2ox7* showed no detectable effect on RPH when mutated, as the *osga2ox7* single mutant was indistinguishable from WT (**Figure 3E**; **Table 2**). However, the height-promoting effects of other class I mutants were attenuated by the presence of *osga2ox7*. For example, the RPH of *osga2ox4* was 108.2%, whereas the *osga2ox4/7* double mutant showed a reduced RPH of 99.3%, representing an 8.9% decrease relative to *osga2ox4* (**Table 2**, **4**). A comparable reduction was observed for *osga2ox7/8* relative to *osga2ox8* (104.6% vs. 95.7%, an 8.9% decrease) (**Table 2**, **4**). Similarly, the RPH of *osga2ox3/4* was 120.6%, but decreased to 111.9% in the *osga2ox3/4/7* triple mutant (an 8.7% reduction), while the RPH of *osga2ox3/8* declined from 111.6% to 101.4% in *osga2ox3/7/8* (a 10.2% reduction) (**Table 4**). This suppressive effect of *osga2ox7* on plant height is consistent with a previous report in which *osga2ox3/7* double mutants were slightly shorter than *osga2ox3* alone (Jin et al., 2023). Moreover, pairwise statistical comparisons supported a suppressive effect of *osga2ox7* in double and triple mutant backgrounds, suggesting that *OsGA2ox7* can partially counteract the growth-promoting effects of class I *OsGA2ox* disruption. Notably, this interaction was not evident in the quadruple mutant (**Supplementary Figure S4C**). Consistent with these observations, factorial linear model analysis revealed significant negative interactions between *osga2ox4/7* and *osga2ox7/8* (P < 0.001), indicating suppressive genetic interactions on plant height (**Supplementary Table S2**). Together, these results indicate that *OsGA2ox7* exhibits a context-dependent interaction pattern, with phenotypic effects that become apparent only when *osga2ox7* is combined with other *OsGA2ox* mutations.

Given this unusual interaction pattern, the mechanism underlying the plant height suppression effect of *osga2ox7* remains unclear. Previous work showed that *OsGA2ox7*-overexpressing lines accumulated only ~30% of WT GA_1_ and ~60% of WT GA_4_ levels (Jin et al., 2023), indicating preferential activity in the 13-hydroxylation pathway. Consequently, loss of *OsGA2ox7* may redirect GA biosynthesis toward this pathway, ultimately reducing plant height. A similar shift in GA flux has been observed in Arabidopsis overexpressing *OsGA13ox*, which drives GA metabolism toward the 13-hydroxylation branch and produces semi-dwarf phenotypes (Magome et al., 2013). Thus, altered flux between the 13-hydroxylation and non-13-hydroxylation GA pathways provides a plausible explanation.

#### 4.2.4 *OsGA2ox7* and *OsGA2ox8* modulate thousand-grain weight

Previous studies have implicated GA in the regulation of grain weight, particularly through GA-responsive genes such as *OsGASR9* and *GW6*, which influence grain size and yield (Li et al., 2019; Shi et al., 2020). Excessive GA signaling has also been reported to reduce grain weight and yield (Kwon et al., 2015). These findings suggest that GA availability during grain development is a key determinant of TGW.

Consistent with this view, we observed reductions in TGW in multiple mutants, including *osga2ox4/7, osga2ox7/8*, *osga2ox3/7/8*, *osga2ox4/7/8*, and *osga2ox3/4/7/8*, with values ranging from 21.1 g to 22.4 g (85–90% of WT; **Table 4**). The reduction was most pronounced when *OsGA2ox7* and *OsGA2ox8* were simultaneously disrupted, indicating that their combined loss primarily drives this effect. However, this hypothesis requires further validation through direct hormone quantification.

### 4.3 Functional roles of class III *GA2ox* genes in rice

In rice, class III *GA2ox* genes form a distinct subgroup of C20-type enzymes that includes *OsGA2ox5*, *OsGA2ox6*, and *OsGA2ox9*. *OsGA2ox9* is a duplicate of *OsGA2ox6*, while *OsGA2ox5* arose as a retrogene (Hsieh et al., 2021). Among these, *OsGA2ox6* shows relatively higher expression in seeds, panicles, and shoots, whereas *OsGA2ox5* is predominantly expressed in roots, and *OsGA2ox9* is barely detectable in most tissues (Hsieh et al., 2021). *OsGA2ox5 OsGA2ox6* and *OsGA2ox9* did not show clear responsiveness to GA treatment (**Supplementary Figure S5**). Although *OsGA2ox6* has been proposed as the dominant paralog (Hsieh et al., 2021), the relative importance of these class III genes in rice development has not been fully resolved.

#### 4.3.1 *OsGA2ox6* as the predominant class III regulator of plant height

In this study, the *osga2ox6* mutant displayed the most pronounced increase in plant height, with RPH approximately 15% taller than WT (**Figures 5C, D**; **Table 2**). By contrast, the *osga2ox5* mutant showed only a moderate increase of about 5% (**Figures 5A, B**; **Table 2**), while *osga2ox9* had no significant effect (**Figures 5E, F**; **Table 2**). These results highlight differential contributions of the three class III genes to plant height regulation.

However, reports from earlier studies have been inconsistent. Knockout of all three genes was initially described as having no effect (Chen et al., 2019). Similarly, the *osga2ox9* mutant showed no visible effect in plant height (Xing et al., 2023). In contrast, later work demonstrated that *osga2ox6* mutants were taller (He et al., 2024). Our findings align with this later observation, confirming *OsGA2ox6* as the predominant class III regulator of plant height, while *OsGA2ox5* playing a smaller but measurable role. Importantly, the consistent increase in plant height across four independent *osga2ox6* knockout alleles in our study underscores the robustness of this conclusion and provides strong evidence that the effect is general rather than background- or allele-specific (**Figure 5C**).

#### 4.3.2 *OsGA2ox6* modulates grain number and seed size

A previous study demonstrated that loss-of-function mutations in *OsGA2ox5* and *OsGA2ox6* influence seed size, seed quality, and fertility, emphasizing the non-redundant roles of these paralogs in rice development (Chen et al., 2019). In this study, knockouts of both *OsGA2ox5* and *OsGA2ox6* reduced yield per plant (YPP), primarily through a decrease in grain number per panicle (GNP), with *osga2ox5* showing a more pronounced reduction and lower fertility overall (**Table 2**). By contrast, although YPP was reduced in *osga2ox6*, this mutant exhibited a significant increase in thousand-grain weight (TGW), attributable to larger seeds mainly resulting from increased seed length (**Figure 6**; **Table 2**). These findings point to a trade-off mediated by *OsGA2ox6* between grain number and seed size.

Principal component analysis of the phenotypic dataset further supported this distinctive behavior. Among the class III mutants, *osga2ox6* showed partial separation from *osga2ox5* and *osga2ox9* and was positioned in association with traits related to plant growth and grain size, including relative plant height (RPH), thousand-grain weight (TGW), and seed setting rate. In contrast, *osga2ox5* and *osga2ox9* were more closely associated with yield-related traits such as panicle number (PN), grain number per panicle (GNP), and yield per plant (YPP) (**Supplementary Figure S6**). Although this separation was moderate, it is consistent with the phenotypic analyses and highlights the distinct contribution of *OsGA2ox6* to growth- and seed-size–related traits.

Interestingly, previous studies in Arabidopsis and legumes have shown a general correlation between seed size and dormancy, with larger seeds typically associated with reduced dormancy (Rubio de Casas et al., 2017; Krzyszton et al., 2024). Consistent with this trend, both *OsGA2ox6* and *OsGA2ox9* have been reported to promote seed dormancy (Xing et al., 2023; Shen et al., 2024), yet only *osga2ox6* knockouts displayed increased seed size in our study (**Figure 6**). Although *OsGA2ox6* and *OsGA2ox9* are paralogs (Hsieh et al., 2021), their effects on seed size and dormancy point to functional divergence following duplication, likely driven by differences in tissue-specific expression and *GA2ox* enzymatic activity (Lo et al., 2008; Chen et al., 2019; Hsieh et al., 2021).

Together, these results refine previous conclusions by providing allele-validated evidence in a uniform genetic background. While both *OsGA2ox5* and *OsGA2ox6* clearly influence yield and fertility, *OsGA2ox6* emerges as a key regulator linking grain yield, seed size, and dormancy, highlighting its distinct role among class III paralogs and underscoring the evolutionary divergence within this gene family.

#### 4.3.3 *OsGA2ox6* modulates flowering time

Among the *ga2ox* knockout mutants, *OsGA2ox6* had the strongest effect on heading date, with *osga2ox6* plants flowering more than four days earlier than the wild type (83.1 vs. 87.9 DAI; **Table 2**). This contrasts with the *Dap1* mutant, in which elevated *OsGA2ox6* expression delays flowering by 16 days (Tang et al., 2023). These opposite phenotypes point to a role for *OsGA2ox6* in maintaining GA homeostasis, which, in turn, influences heading date.

From an evolutionary perspective, GAs are considered important regulators of sexual reproduction in vascular plants since the emergence of the GA-GID1-DELLA signaling module (Zhao et al., 2025). Later in angiosperm lineages, class III GA 2-oxidases arose as additional layers of control (Yoshida et al., 2020). Among them, *OsGA2ox6* stands out because its expression is strongly induced by abiotic stress and stress-related phytohormones (Yoshida et al., 2020). This suggests that genes such as *OsGA2ox6* act as environmental integrators, feeding external cues into the GA-GID1-DELLA regulatory network. By doing so, they fine-tune reproductive timing in angiosperms, ensuring proper flowering under fluctuating environmental conditions.

### 4.4 Additional insight into *GA2ox* function during seed germination

Given the well-established role of GA in promoting seed germination, we examined whether disruption of *OsGA2ox* genes affects germination behavior. Germination assays were conducted using representative mutants from different classes (*osga2ox1*, *osga2ox3*, and *osga2ox4*) in comparison with the WT.

Unexpectedly, all examined mutants exhibited delayed rather than accelerated germination (**Supplementary Figure S7**). This observation suggests that loss of OsGA2ox function does not simply enhance GA-mediated germination, but instead disrupts the proper regulation of GA homeostasis during early seed imbibition.

In our previous study, most *OsGA2ox* genes were shown to be expressed upon seed imbibition (Lo et al., 2008), indicating that these genes may play a role in fine-tuning GA levels during germination. Therefore, the delayed germination observed in the mutants might have resulted from imbalanced or misregulated GA levels, rather than from increased GA activity.

## 5 Summary

**Figure 7:**
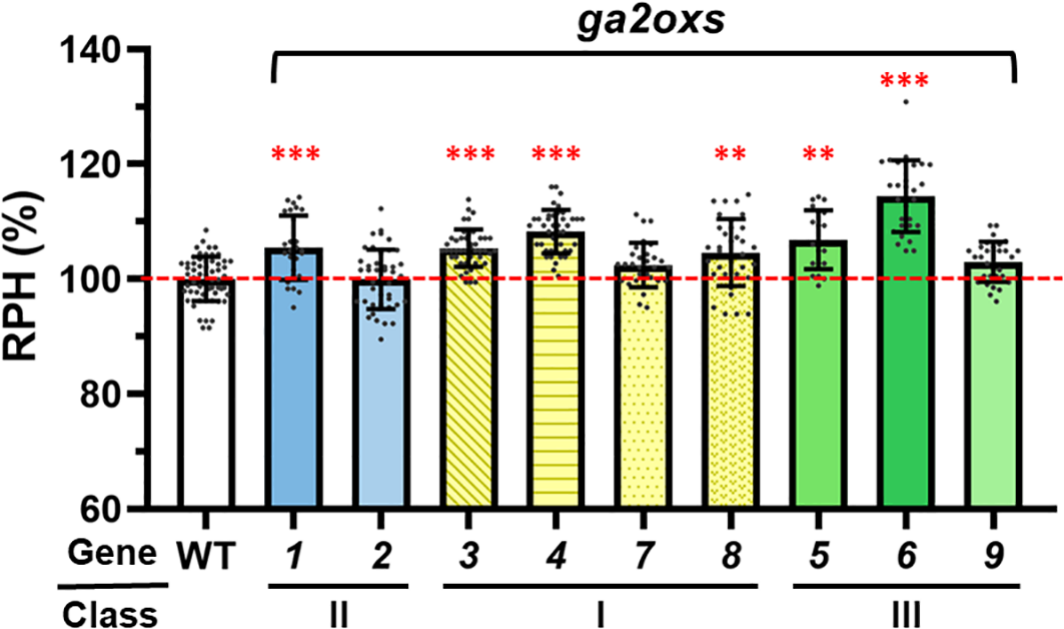
Functional hierarchy of *osga2ox* genes on plant height regulation. Relative plant heights (RPH, %) of the wild type (WT) and independent mutants of *osga2ox* paralogs from Class II (*osga2ox1, osga2ox2*), Class I (*osga2ox3, osga2ox4, osga2ox7, osga2ox8*), and Class III (*osga2ox5, osga2ox6, osga2ox9*). Bars represent means ± SE. The red dashed line marks the WT average. The statistical significance compared with WT was determined by one-way ANOVA followed by Tukey’s test; **P < 0.01, ***P < 0.001.

Taken together, our study defines a functional hierarchy within the rice *GA2ox* gene family (**Figure 7**). *OsGA2ox1* (class II), *OsGA2ox3* and *OsGA2ox4* (class I), and *OsGA2ox6* (class III) emerge as the dominant regulators of rice development. Other paralogs contribute subsidiary or context-dependent effects, reflecting both redundancy and specialization across classes. By providing allele-validated evidence in a uniform genetic background, our findings clarify the functional divergence of *GA2ox* paralogs and highlight the value of systematic family-wide analyses for resolving dominant versus minor roles.

Although GA2ox enzymes are known to deactivate bioactive GAs, direct measurement of GA levels was not performed in this study. Therefore, our conclusions regarding GA homeostasis were based on phenotypic inference. Future studies incorporating hormone quantification will be required to validate these findings.

## Data Availability

The data presented in this study are included in the article and supplementary material. Further inquiries can be directed to the corresponding author.

## Supplementary material

Supplementary Figure S1 Structure of *OsGA2ox* genes and their CRISPR/Cas9 target sites. **(A–C)** Schematic diagrams to show the *OsGA2ox* Class II **(A)**, Class I **(B)**, and Class III **(C)** genes, and their CRISPR/Cas9 target sites with detailed sgRNA sequences, locations of primers and sizes of PCR products. Exons are shown as yellow boxes with numbers indicating sequence length (bp), and introns are shown as black lines. Blue arrows indicate the sgRNA target sites, with the corresponding sgRNA sequences shown in the box. Positions of PCR primers (black arrows) and the expected PCR fragment sizes (bp) are indicated. **(D)** Schematic diagram of the template CRISPR/Cas9 construct used for transformation, containing the Cas9 coding sequence driven by the maize ubiquitin promoter, the gRNA scaffold under the control of the rice U3 promoter, and the hygromycin resistance gene (*hpt*) under the CaMV 35S promoter.

Supplementary Figure S2-1 Schematic diagram to describe the screening and selection process for gene-edited *osga2oxs* mutation variants. The schematic workflow shows the generation and screening of *osga2oxs* CRISPR/Cas9-edited rice plants. Wild-type calli were transformed, and regenerated T0 plants were analyzed by PCR and sequencing for T-DNA construct (Cnt) presence and target site mutations. Gene-edited T1 plants were classified into homozygous, heterozygous/biallelic, and non-edited categories, each with or without the Cnt. During T2 generation selection, non-edited plants and heterozygous lines carrying the Cnt were eliminated. Successive generations (T2–T6) were propagated and analyzed by PCR and sequencing to monitor allele segregation and remove the Cnt. This process yielded stable homozygous A- and B-variants, including Cnt-free lines suitable for downstream functional studies.

Supplementary Figure S2-2 Variant screening and selection at T1 generation. The *osga2ox4* variant line #10.1 is used as example. **(A)** PCR and sequencing analysis of T1 plants from *ga2ox4-10.1*. The top panel shows detection of the T-DNA construct, and the bottom panel shows an analysis of target site mutations. Multiple band patterns represent biallelic variants, while single bands correspond to homozygous or wild-type alleles. Plants with −38 bp and −2 bp alleles were identified, either with or without the construct. Five plants whose sequences were identified are shown in panel **(B)** and colored in red. **(B)** Sequence alignments of T1 plants relative to the wild-type *OsGA2ox4* sequence. The PAM site is highlighted in grey and the sgRNA sequence in yellow. Line #10.1 produced −38 bp and −2 bp deletions. Among the analyzed plants, plant #13 carried a −38 bp homozygous allele with the T-DNA construct removed and was selected for further propagation. In contrast, plant #5 carried a −38 bp homozygous allele with the construct, and plants #15 and #25 carried −2 bp homozygous alleles with the construct. Plant #21 represented a bi-allelic edit variant without the construct and will be propagated to the next generation to get the -2 and -38 homo-allelic plants.

Supplementary Figure 3-1 PCR validation, sequence analysis, and amino acid changes of *osga2ox1* variants generated by CRISPR/Cas9. **(A)** PCR amplification of the *OsGA2ox1* target region in wild type (WT) and representative variant lines (2.1, 5.3, and 5.4). Band sizes correspond to WT (594 bp) and insertion or deletion (In/Del) variants: +1 (595 bp), −84 (510 bp), and −42 (552 bp). **(B)** DNA sequence alignments of *OsGA2ox1* in WT and variants. The sgRNA sequence (yellow) and PAM (grey) are highlighted. Variant alleles show an insertion (+1) or two deletions (−84, −42) at the target site, generating In/Del polymorphisms. **(C)** Amino acid sequence comparisons of *OsGA2ox1* in WT and variants. Predicted protein products reveal altered ORFs due to In/Del-induced frameshift mutations, disrupting the conserved domains DIOX_N and 2OG-FeII_Oxy. Functional motifs (yellow and green) are either disrupted or lost, and regions with disrupted ORFs are highlighted in red.

Supplementary Figure S3-2 PCR validation, sequence analysis, and amino acid changes of *osga2ox2* variants generated by CRISPR/Cas9. **(A)** PCR amplification of the *OsGA2ox2* target region in wild type (WT) and representative variant lines (2.1, 3.1, and 3.2). Band sizes correspond to WT (362 bp or 1589 bp) and insertion or deletion (In/Del) variants: +1 (363 bp), −35 (327 bp) and −738 (851 bp). **(B)** DNA sequence alignments of *OsGA2ox2* in WT and variants. The sgRNA sequence (yellow) and PAM (grey) are highlighted. Variant alleles show an insertion (+1) or two deletions (−35, −738) at the target site, generating In/Del polymorphisms. **(C)** Amino acid sequence comparisons of *OsGA2ox2* in WT and variants. Predicted protein products reveal altered ORFs due to In/Del-induced frameshift mutations, disrupting the conserved domains DIOX_N and 2OG-FeII_Oxy. Functional motifs (yellow and green) are either disrupted or lost, and regions with disrupted ORFs are highlighted in red.

Supplementary Figure S3-3 PCR validation, sequence analysis, and amino acid changes of *osga2ox3* variants generated by CRISPR/Cas9. **(A)** PCR amplification of the *OsGA2ox3* target region in wild type (WT) and representative variant lines (4.1, 7.1, 7.2, and 8.2). Band sizes correspond to WT (504 bp) and insertion or deletion (In/Del) variants: +1 (505 bp), −2 (502 bp), −3 (501 bp), and −13 (491 bp). Bi-allelic variants (−2/+1, −13/+1) are also detected. **(B)** DNA sequence alignments of *OsGA2ox3* in WT and variants. The sgRNA sequence (yellow) and PAM (grey) are highlighted. Variant alleles show an insertion (+1) or three deletions (−2, −3, −13) at the target site, as well as bi-allelic combinations, generating In/Del polymorphisms. **(C)** Amino acid sequence comparisons of *OsGA2ox3* in WT and variants. Predicted protein products reveal altered ORFs due to In/Del-induced frameshift mutations, disrupting the conserved domains DIOX_N and 2OG-FeII_Oxy. Functional motifs (yellow and green) are either disrupted or lost, and regions with disrupted ORFs are highlighted in red.

Supplementary Figure S3-4 PCR validation, sequence analysis, and amino acid changes of *osga2ox4* variants generated by CRISPR/Cas9. **(A)** PCR amplification of the *OsGA2ox4* target region in wild type (WT) and representative variant lines (1.1, 2.1, 3.1, and 10.1). Band sizes correspond to WT (488 bp) and insertion or deletion (In/Del) variants: +1 (489 bp), −2 (486 bp), and −38 (450 bp). **(B)** DNA sequence alignments of *OsGA2ox4* in WT and variants. The sgRNA sequence (yellow) and PAM (grey) are highlighted. Variant alleles show an insertion (+1) or two deletions (−2, −38) at the target site, generating In/Del polymorphisms. **(C)** Amino acid sequence comparisons of *OsGA2ox4* in WT and variants. Predicted protein products reveal altered ORFs due to In/Del-induced frameshift mutations, disrupting the conserved domains DIOX_N and 2OG-FeII_Oxy. Functional motifs (yellow and green) are either disrupted or lost, and regions with disrupted ORFs are highlighted in red.

Supplementary Figure S3-5 PCR validation, sequence analysis, and amino acid changes of *osga2ox5* variants generated by CRISPR/Cas9. **(A)** PCR amplification of the *OsGA2ox5* target region in wild type (WT) and representative variant lines (4.1, 9.1, 9.2, and 10.2). Band sizes correspond to WT (467 bp) and insertion or deletion (In/Del) variants: +1 (468 bp), −7 (460 bp), −12 (455 bp), and −48 (419 bp). **(B)** DNA sequence alignments of *OsGA2ox5* in WT and variants. The sgRNA sequence (yellow) and PAM (grey) are highlighted. Variant alleles show an insertion (+1) or deletions (−7, −12, −48) at the target site, generating In/Del polymorphisms. **(C)** Amino acid sequence comparisons of OsGA2ox5 in WT and variants. Predicted protein products reveal altered ORFs due to In/Del-induced frameshift mutations, disrupting the conserved domains DIOX_N and 2OG-FeII_Oxy. Functional motifs (yellow and green) are either disrupted or lost, and regions with disrupted ORFs are highlighted in red.

Supplementary Figure S3-6 PCR validation, sequence analysis, and amino acid changes of *osga2ox6* variants generated by CRISPR/Cas9. **(A)** PCR amplification of the *OsGA2ox6* target region in wild type (WT) and representative variant lines (1.1, 1.2, 4.2, and 6.2). Band sizes correspond to WT (461 bp) and insertion or deletion (In/Del) variants: +1 (462 bp), −7 (454 bp), −31 (430 bp), and −91 (370 bp). Bi-allelic variants (−7/+1) are also detected. **(B)** DNA sequence alignments of *OsGA2ox6* in WT and variants. The sgRNA sequence (yellow) and PAM (grey) are highlighted. Variant alleles show an insertion (+1) or three deletions (−7, −31, −91) at the target site, as well as bi-allelic combinations (−7/+1), generating In/Del polymorphisms. **(C)** Amino acid sequence comparisons of OsGA2ox6 in WT and variants. Predicted protein products reveal altered ORFs due to In/Del-induced frameshift mutations, disrupting the conserved domains DIOX_N and 2OG-FeII_Oxy. Functional motifs (yellow and green) are either disrupted or lost, and regions with disrupted ORFs are highlighted in red.

Supplementary Figure S3-7 PCR validation, sequence analysis, and amino acid changes of *osga2ox7* variants generated by CRISPR/Cas9. **(A)** PCR amplification of the *OsGA2ox7* target region in wild type (WT) and representative variant lines (5.1, 6.1, 8.1, and 10.1). Band sizes correspond to WT (334 bp) and insertion or deletion (In/Del) variants: −5 (329 bp), +43 (377 bp), +1 (335 bp), and −2 (332 bp). **(B)** DNA sequence alignments of *OsGA2ox7* in WT and variants. The sgRNA sequence (yellow) and PAM (grey) are highlighted. Variant alleles show insertions (+1, +43) and deletions (−2, −5) at the target site, generating In/Del polymorphisms. **(C)** Amino acid sequence comparisons of OsGA2ox7 in WT and variants. Predicted protein products reveal altered ORFs due to In/Del-induced frameshift mutations, disrupting the conserved domains DIOX_N and 2OG-FeII_Oxy. Functional motifs (yellow and green) are either disrupted or lost, and regions with disrupted ORFs are highlighted in red.

Supplementary Figure S3-8 PCR validation, sequence analysis, and amino acid changes of *osga2ox8* variant generated by CRISPR/Cas9. **(A)** PCR amplification of the *OsGA2ox8* target region in wild type (WT) and representative variant lines (1.4, 2.1, 5.1, and 9.1). Band sizes correspond to WT (398 bp) and insertion or deletion (In/Del) variants: −19 (379 bp), +1 (399 bp), −33 (365 bp), and −41 (357 bp). **(B)** DNA sequence alignments of *OsGA2ox8* in WT and variants. The sgRNA sequence (yellow) and PAM (grey) are highlighted. Variant alleles show an insertion (+1) or three deletions (−19, −33, −41) at the target site, generating In/Del polymorphisms. **(C)** Amino acid sequence comparisons of OsGA2ox8 in WT and variants. Predicted protein products reveal altered ORFs due to In/Del-induced frameshift mutations, disrupting the conserved domains DIOX_N and 2OG-FeII_Oxy. Functional motifs (yellow and green) are either disrupted or lost, and regions with disrupted ORFs are highlighted in red.

Supplementary Figure S3-9 PCR validation, sequence analysis, and amino acid changes of *osga2ox9* variants generated by CRISPR/Cas9. **(A)** PCR amplification of the *OsGA2ox9* target region in wild type (WT) and representative variant lines (8.1, 11.1, and 11.2). Band sizes correspond to WT (327 bp) and insertion or deletion (In/Del) variants: +25 (352 bp), −2 (325 bp), −5 (322 bp), −21 (306 bp), -55 (272 bp). Bi-allelic variants (−55/−21) are also detected. **(B)** DNA sequence alignments of *OsGA2ox9* in WT and variants. The sgRNA sequence (yellow) and PAM (grey) are highlighted. Variant alleles show an insertion (+25) or deletions (−2, -5, −21, −55), as well as bi-allelic combinations (−55/−21), generating In/Del polymorphisms. **(C)** Amino acid sequence comparisons of OsGA2ox9 in WT and variants. Predicted protein products reveal altered ORFs due to In/Del-induced frameshift mutations, disrupting the conserved domains DIOX_N and 2OG-FeII_Oxy. Functional motifs (yellow and green) are either disrupted or lost, and regions with disrupted ORFs are highlighted in red.

Supplementary Figure S4 Interaction effects among Class I *ga2ox* mutants revealed by pairwise comparisons. Relative plant height (RPH) data from available single- and multiple-gene knockout combinations were compared with *ga2ox3* **(A)**, *ga2ox4* **(B)**, *ga2ox7* **(C)**, and *ga2ox8* **(D)** mutants using the nonparametric Mann–Whitney test. *P*-values (**p* < 0.05, ***p* < 0.01, ****p* < 0.001) are shown in the corresponding boxes and color-coded to indicate additive effects (red), suppressive effects (green), or no detectable effects (gray). NA indicates data not available.

Supplementary Figure S5 Expression profiles of *OsGA2ox* genes in response to GA treatment. Expression profiles of *OsGA2ox* genes from Class I (*OsGA2ox3*, *OsGA2ox4*, *OsGA2ox7*, and *OsGA2ox8*), Class II (*OsGA2ox1* and *OsGA2ox2*), and Class III (*OsGA2ox5*, *OsGA2ox6*, and *OsGA2ox9*) following GA treatment were obtained from the RiceXPro database. Seven-day-old rice seedlings (Oryza sativa L. japonica cv. Nipponbare) were treated with 10 μM GA_3_, and shoot samples were collected at 0, 1, 3, 6, and 12 hours after treatment. Expression levels are presented as signal intensity values. Green bars represent control samples, and red bars represent GA-treated samples. Two biological replicates are shown for each time point. Sato Y, Takehisa H, Kamatsuki K, Minami H, Namiki N, Ikawa H, Ohyanagi H, Sugimoto K, Antonio B, Nagamura Y (2013) RiceXPro Version 3.0: expanding the informatics resource for rice transcriptome Nucleic Acids Research 41:D1206-D1213

Supplementary Figure S6 Principal component analysis (PCA) of phenotypic traits in *OsGA2ox* mutant classes. Principal component analysis (PCA) of phenotypic traits was performed for **(A)** Class I (*osga2ox3* and *osga2ox4*, *osga2ox7* and *osga2ox8*), **(B)** Class II (*osga2ox1* and *osga2ox2*), and **(C)** Class III (*osga2ox5*, *osga2ox6*, and *osga2ox9*) mutants. Each point represents an individual plant, and colored symbols indicate different mutant genotypes. Ellipses represent the distribution of each genotype group.

Supplementary Figure S7 Germination rates of WT and *osga2ox* mutants. Seeds from WT and *osga2ox1*, *osga2ox3*, and *osga2ox4* mutants were germinated on MS medium and incubated in a growth chamber at 28 °C under a 16 h light/8 h dark photoperiod. Germination rates were recorded over 6 days after imbibition (DAI). Data represent the mean ± SD from three independent experiments, each consisting of 10 seeds (total n = 30 per genotype).
