# Supplementary material for: Systematic mutagenesis reveals dominant–minor paralog configurations in the rice GA2ox gene family
Source: Front Plant Sci. 2026 May 7;17:1813123. doi: 10.3389/fpls.2026.1813123 (PMC13190458; doi:10.3389/fpls.2026.1813123)
Supplement: Supplementary Figure S1 — Structure of OsGA2ox genes and their CRISPR/Cas9 target sites. (A–C) Schematic diagrams to show the OsGA2ox Class II (A), Class I (B), and Class III (C) genes, and their CRISPR/Cas9 target sites with detailed sgRNA sequences, locations of primers and sizes of PCR products. Exons are shown as yellow boxes with numbers indicating sequence length (bp), and introns are shown as black lines. Blue arrows indicate the sgRNA target sites, with the corresponding sgRNA sequences shown in the box. Positions of PCR primers (black arrows) and the expected PCR fragment sizes (bp) are indicated. (D) Schematic diagram of the template CRISPR/Cas9 construct used for transformation, containing the Cas9 coding sequence driven by the maize ubiquitin promoter, the gRNA scaffold under the control of the rice U3 promoter, and the hygromycin resistance gene (hpt) under the CaMV 35S promoter. [file DataSheet1.pdf]

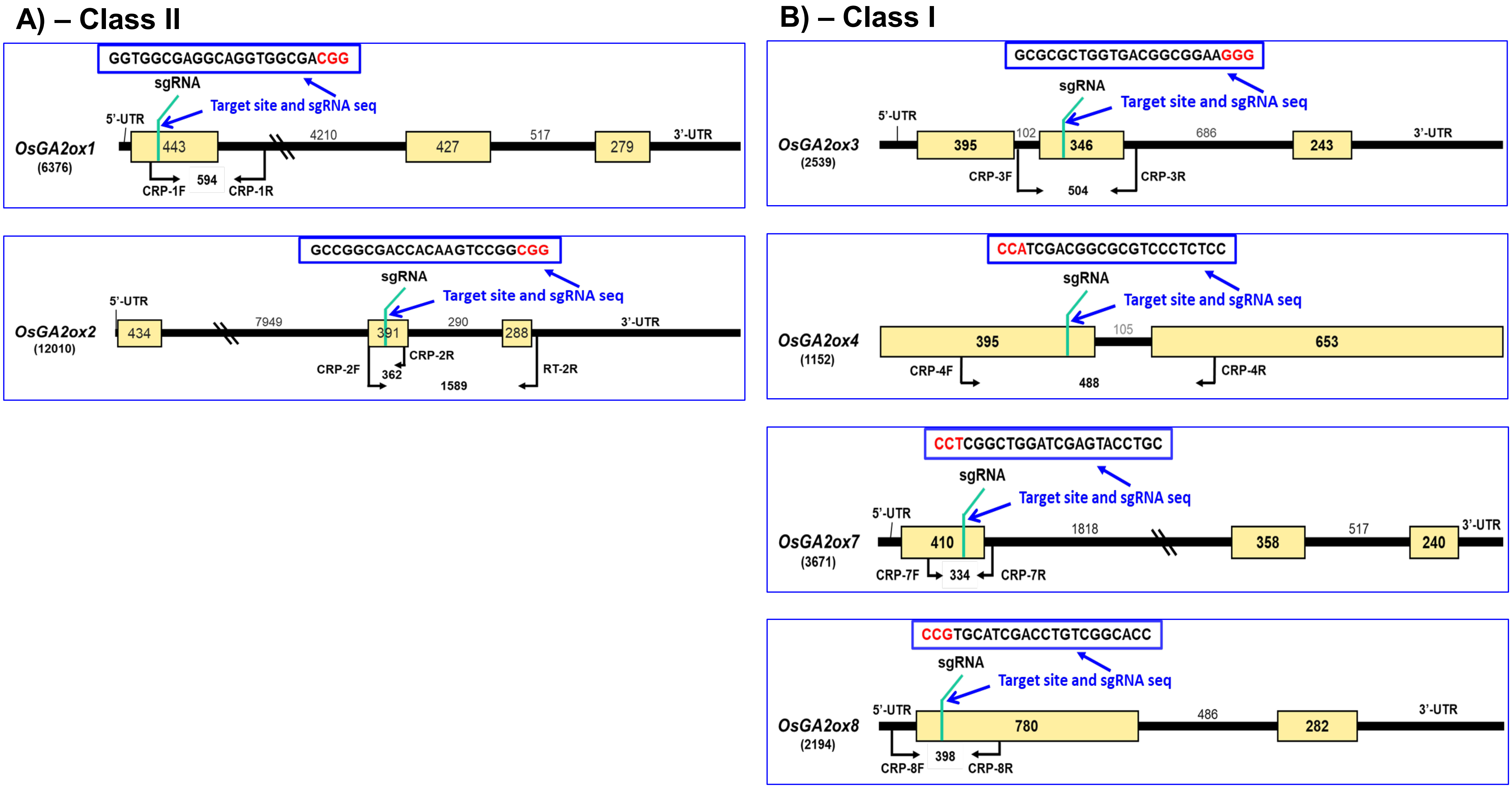

Fig S1-1: Diagrams to show all *OsGA2ox* gene CRISPR/Cas9 constructs with detailed sgRNA seq, target sites, locations of primers and sizes of PCR products

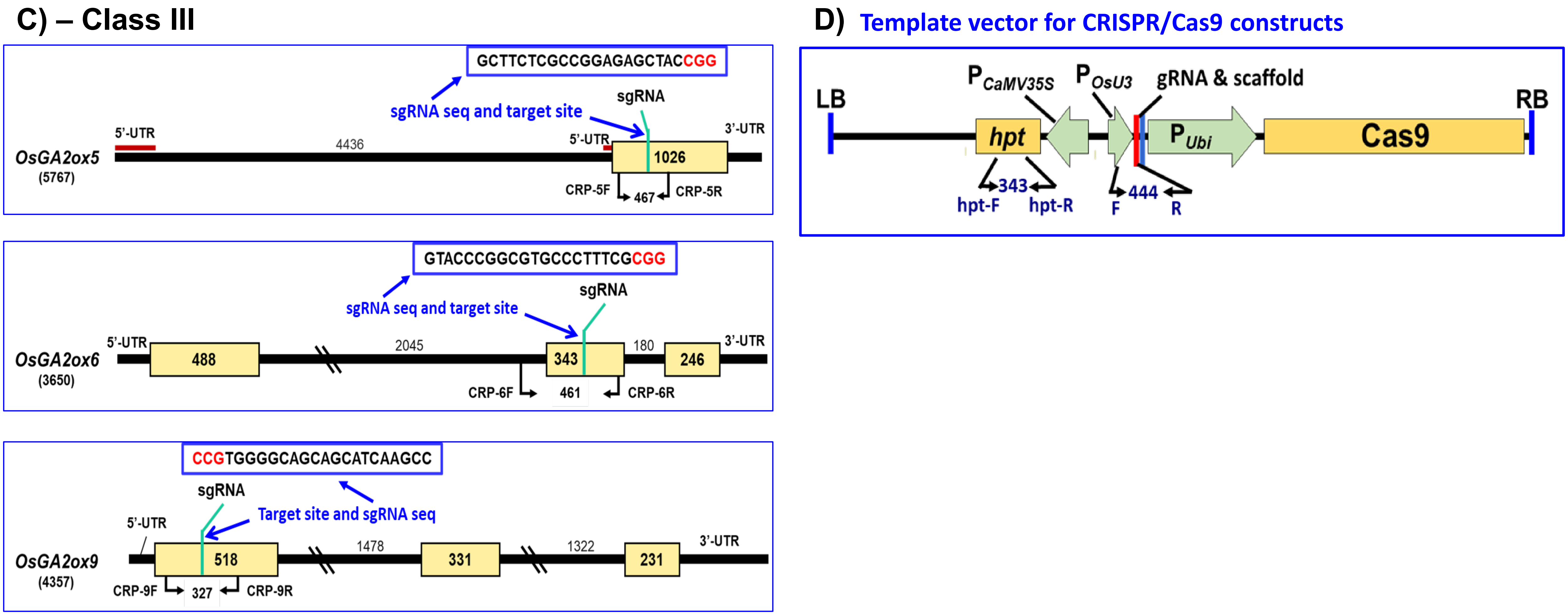

Fig S1-2. Diagrams to show all *OsGA2ox* gene CRISPR/Cas9 constructs with detailed sgRNA seq, target sites, locations of primers and size of PCR products

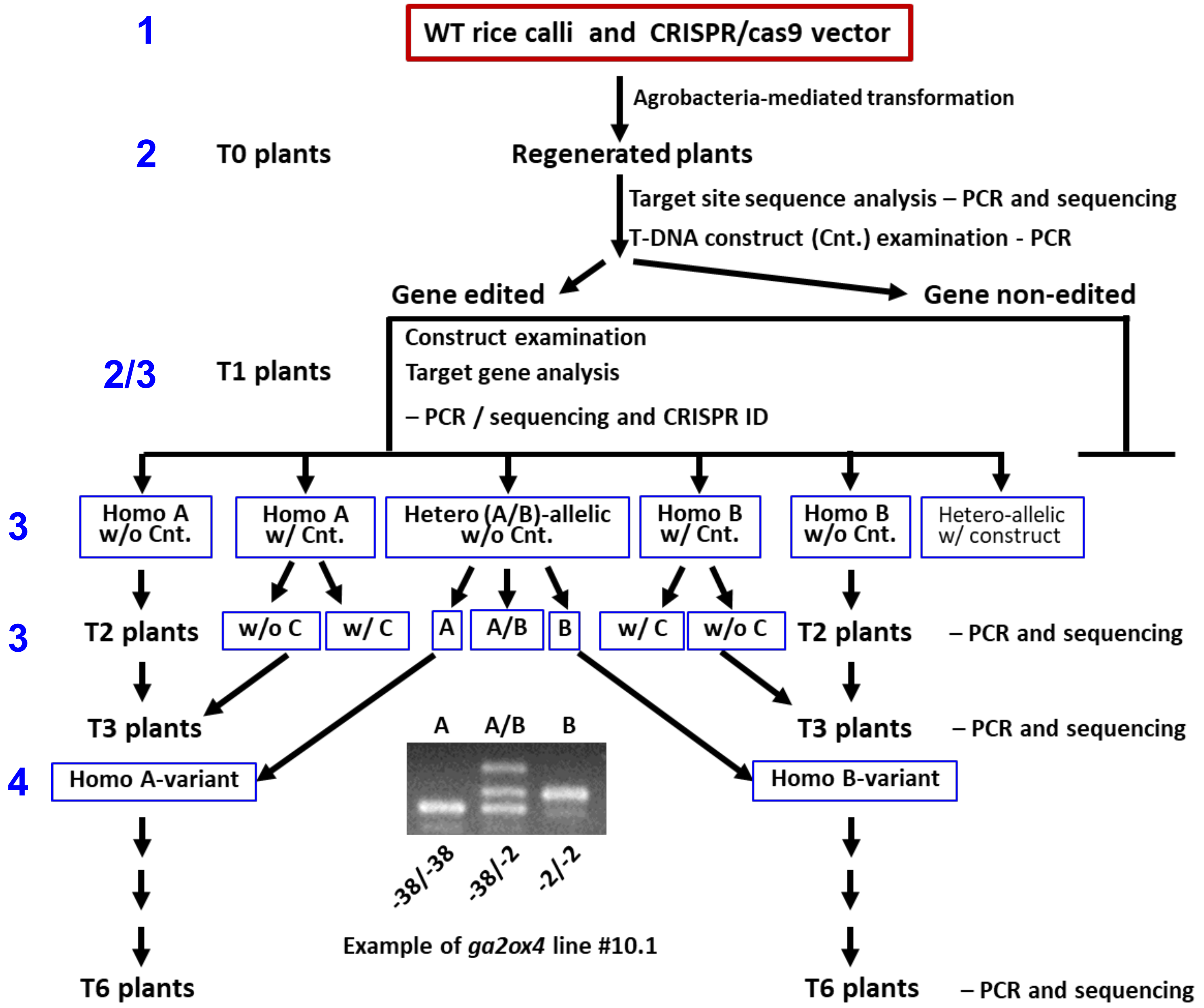

Fig S2-1. An illustration to describe the screening and selection process for gene-edited variants

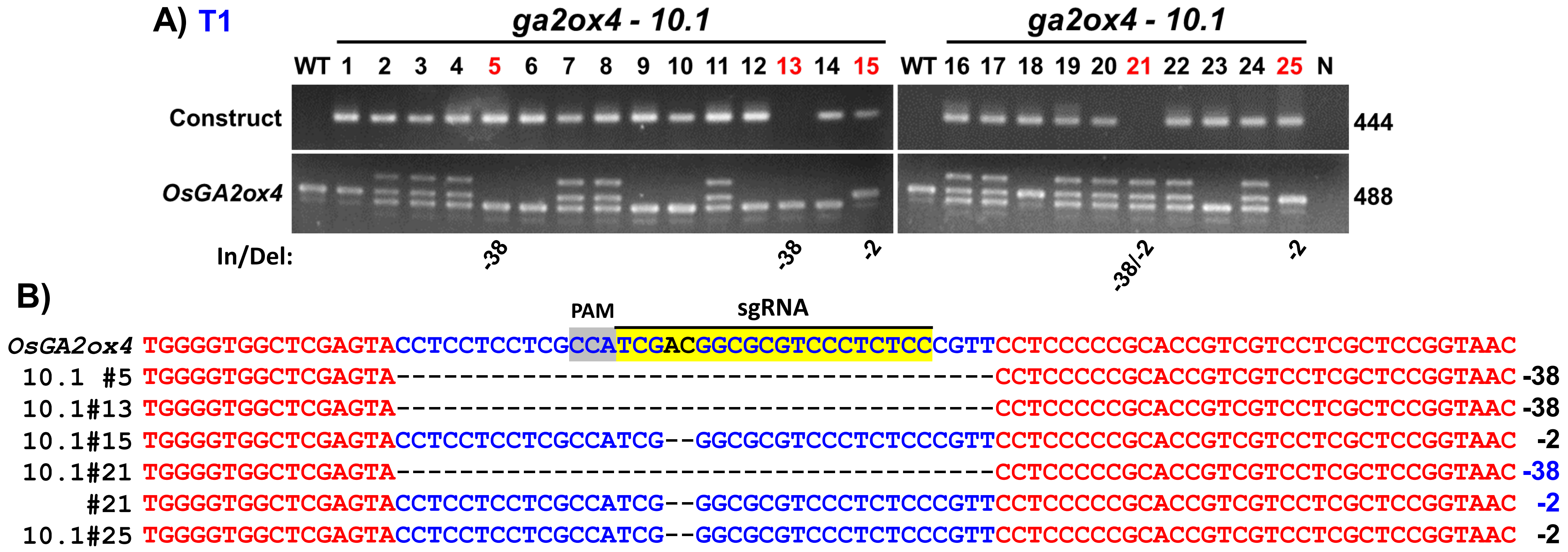

Fig S2-2. Variant screening and selection at T1 generation. The ***osga2ox4*** variant line #10.1 is used as example

At T0 generation, multiple-modifications in line #10.1 were identified and then propagated to the next generation for further selection. In the T1 generation shown here, 25 plants were selected for target site analysis. The T-DNA constructs were examined by PCR and the target regions of *OsGA2ox4* were also PCR amplified and sequenced to confirm their In/Del and allelic types. Luckily, plants with target site modified and T-DNA crossed-out could be obtained at T1 generation (such as plant #13 showing -38 bp modified without T-DNA construct) and progenies of these plants are represented as the -38 bp variant of line #10.1 to be further characterized. Plant #5 is the -38 bp variant with construct and its progenies will be characterized and try to cross out the construct as well. Plants #15 and #25 are the -2 bp variant with construct and their progenies will be characterized and tried to cross out the construct as well. In addition to these homo-alleles, a high proportion of bi-allelic types (pattern of multiple bands) in T1 generation will require further generation to obtain homo-allelic -2 and -38 variants. Plants #21 is the plant of -2/-38 bi-allelic variant without construct and will be propagated to the next generation to get the -2 and -38 homo-allelic plants.

Osga2ox1

A) PCR amplified target DNA and its Seq In/Del in different variants

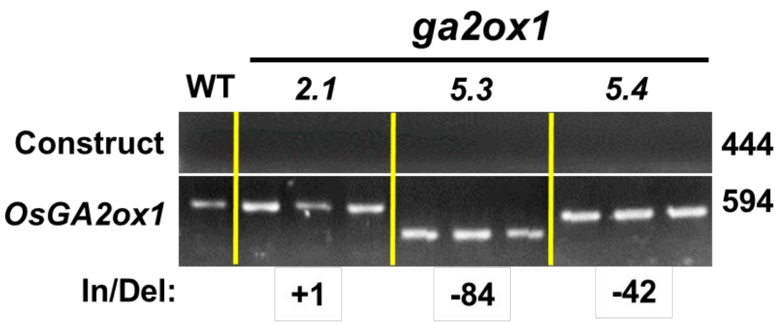

B) DNA seq comparisons

```
                                          sgRNA                PAM
OsGA2ox1         TGTCGGCGGAGCGCGGCGCGGTGGCGAGGCAGGTGG-CGACGGCGTGCGCGGCGCACGGGTTCTTCCGGTGCGTCGGGCACGGCGTGCCGGCGGCGGCGCCCGTCGCGGCGA
#2.1   (+1/+1)   TGTCGGCGGAGCGCGGCGCGGTGGCGAGGCAGGTGGGCGACGGCGTGCGCGGCGCACGGGTTCTTCCGGTGCGTCGGGCACGGCGTGCCGGCGGCGGCGCCCGTCGCGGCGA
#5.3 (-84/-84)   TGTCGGCGGAGCGCGGCGCGGT-------------------------------------------------------------------------------------GGCGA
#5.4 (-42/-42)   TGTCG-------------------------------------------GCGGCGCACGGGTTCTTCCGGTGCGTCGGGCACGGCGTGCCGGCGGCGGCGCCCGTCGCGGCGA
```

C) AA seq comparisons

```
                                          DIOX_N                                        2OG-FeII_Oxy
               10                 40        50        60        70        80        90        100  106            191          310    325   379
OsGA2ox1  MVVPSATTPA[25]TIATVDMSAERGAVARQVATACAAHGFFRCVGHGVPAAAPVAARLDAATAAFFAMAPAEKQRAGPASPLGY[80]NADSDCLLRINHY[110]RPRLSTIYFASPPLHA[53]EGKA* 382
     -84  MVVPSATTPA[25]TIATVDMSAERGAVAR----------------------------LDAATAAFFAMAPAEKQRAGPASPLGY[80]NADSDCLLRINHY[110]RPRLSTIYFASPPLHA[53]EGKA* 354
     -42  MVVPSATTPA[25]TIATVDMSA--------------AHGFFRCVGHGVPAAAPVAARLDAATAAFFAMAPAEKQRAGPASPLGY[80]NADSDCLLRINHY[110]RPRLSTIYFASPPLHA[53]EGKA* 368
      +1  MVVPSATTPA[25]TIATVDMSAERGAVARQVGDGVRGARVLPVRRARRAGGGARRGEAGRRDGGVLRDGAGGEAARRAGEPARVRLPEHRVQRRRRRAGVPAPPRQPRRRRAPGQDHRRHGPLSLQCYCE* 152
```

Fig S3-1. PCR validation, sequence analysis, and amino acid changes of ***osga2ox1*** mutants generated by CRISPR/Cas9

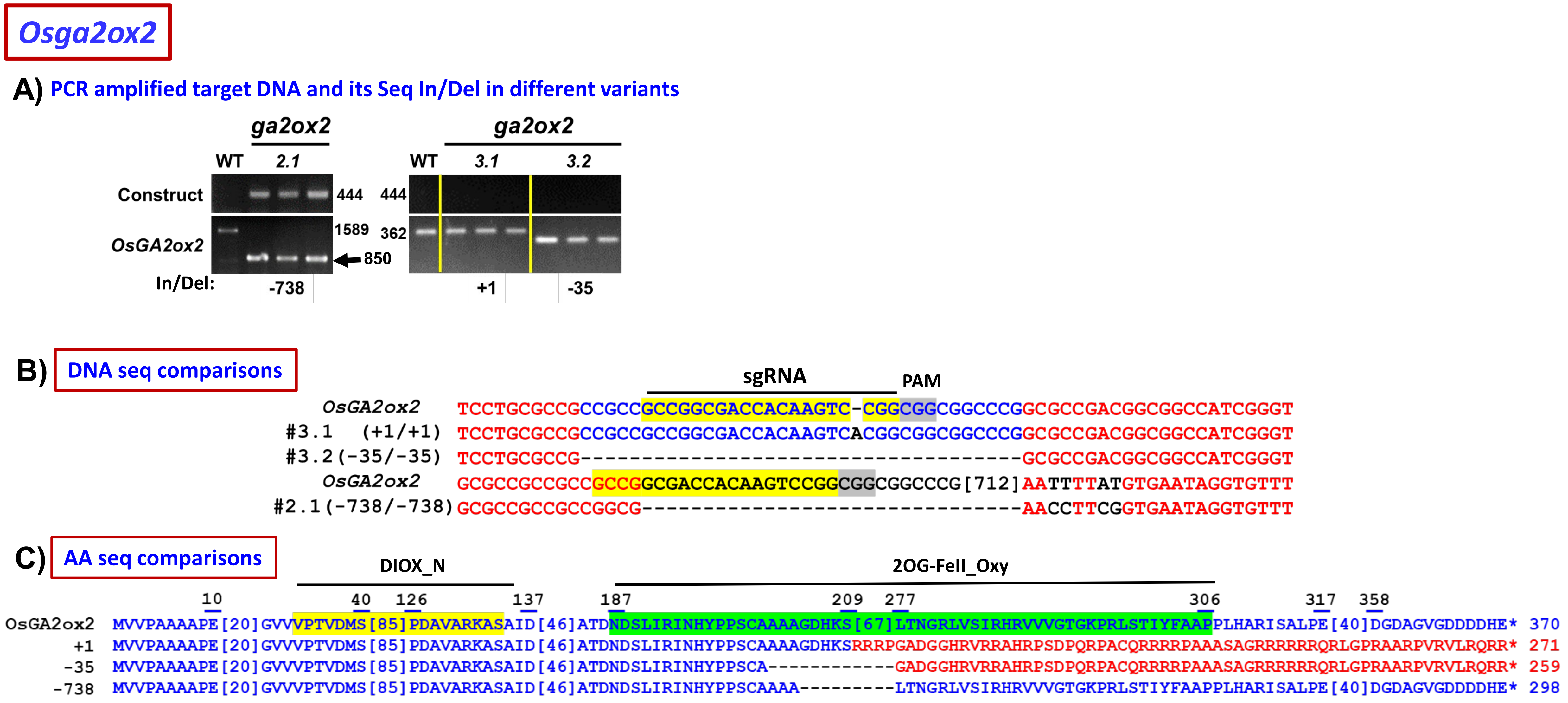

Fig S3-2. PCR validation, sequence analysis, and amino acid changes of ***osga2ox2*** mutants generated by CRISPR/Cas9

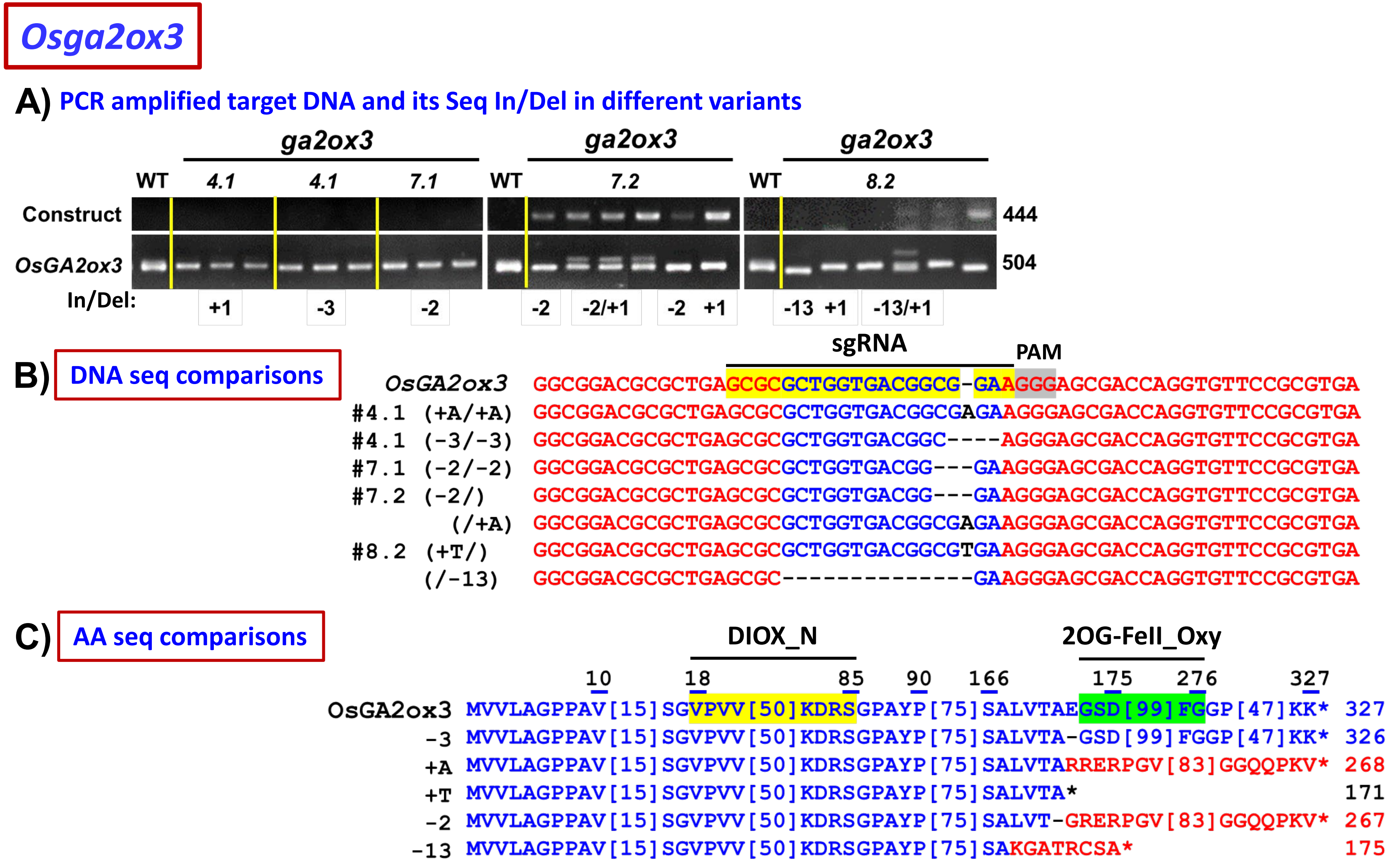

Fig S3-3. PCR validation, sequence analysis, and amino acid changes of ***osga2ox3*** mutants generated by CRISPR/Cas9

Osga2ox4

A) PCR amplified target DNA and its Seq In/Del in different variants

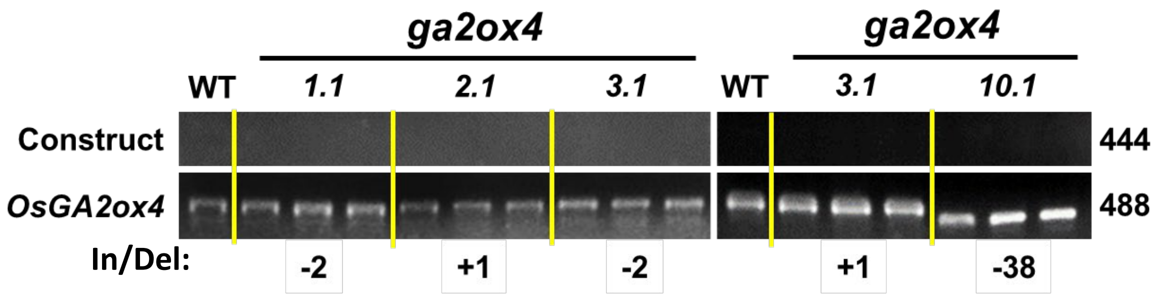

B) DNA seq comparisons

```
                                     PAM        sgRNA
OsGA2ox4        GCTCGAGTACCTCCTCCTCGCCATCG-ACGGCGCGTCCCTCTCCCGTTCCTCCCCCGCACCGTCGTC
#1.1 (-2/-2)    GCTCGAGTACCTCCTCCTCGCCATCG---GGCGCGTCCCTCTCCCGTTCCTCCCCCGCACCGTCGTC
#2.1 (+A/+A)    GCTCGAGTACCTCCTCCTCGCCATCGAACGGCGCGTCCCTCTCCCGTTCCTCCCCCGCACCGTCGTC
#3.1 (-2/-2)    GCTCGAGTACCTCCTCCTCGCCATCG---GGCGCGTCCCTCTCCCGTTCCTCCCCCGCACCGTCGTC
#3.1 (+T/+T)    GCTCGAGTACCTCCTCCTCGCCATCGTACGGCGCGTCCCTCTCCCGTTCCTCCCCCGCACCGTCGTC
#10.1(-38/-38)  GCTCGAGTACCTCC---------------------------------------CCCGCACCGTCGTC
```

C) AA seq comparisons

```
                           DIOX_N                         2OG-FeII_Oxy
         10      29                     127         176          289       301   344
OsGA2ox4 MVVLAKPAAL[15]GAGLPVV[75]LEYLLLAIDGASLSRSSPAPSSSL[40]DGAASDQILRLNH[100]YFGGPPLEQRIAPLRQL[42]RQPPPVANPLA* 354
+A       MVVLAKPAAL[15]GAGLPVV[75]LEYLLLAIERRVPLPFLPRTVVLAPGRGEQ[170]QRRGGGAEPVRGVHVGGVQEGCLPLPPQRQPPGPLPPAATTCR* 350
+T       MVVLAKPAAL[15]GAGLPVV[75]LEYLLLAIVRRVPLPFLPRTVVLAPGRGEQ[170]QRRGGGAEPVRGVHVGGVQEGCLPLPPQRQPPGPLPPAATTCR* 350
-2       MVVLAKPAAL[15]GAGLPVV[75]LEYLLLAI-GRVPLPFLPRTVVLAPGRGEQ[170]QRRGGGAEPVRGVHVGGVQEGCLPLPPQRQPPGPLPPAATTCR* 349
-38      MVVLAKPAAL[15]GAGLPVV[75]LEY-------------LPRTVVLAPGRGEQ[170]QRRGGGAEPVRGVHVGGVQEGCLPLPPQRQPPGPLPPAATTCR* 337
```

Fig S3-4. PCR validation, sequence analysis, and amino acid changes of ***osga2ox4*** mutants generated by CRISPR/Cas9

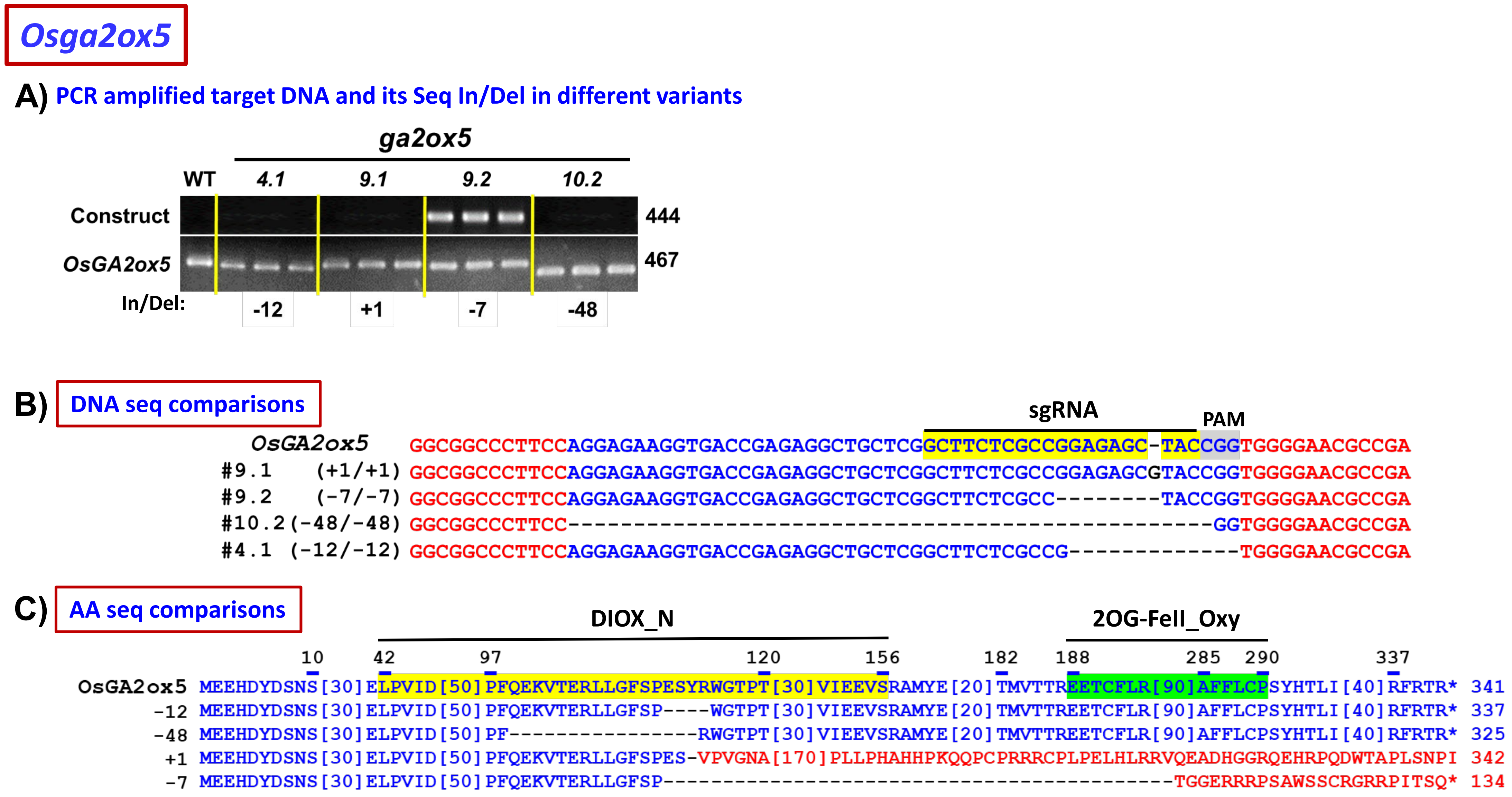

Fig S3-5. PCR validation, sequence analysis, and amino acid changes of ***osga2ox5*** mutants generated by CRISPR/Cas9

Osga2ox6

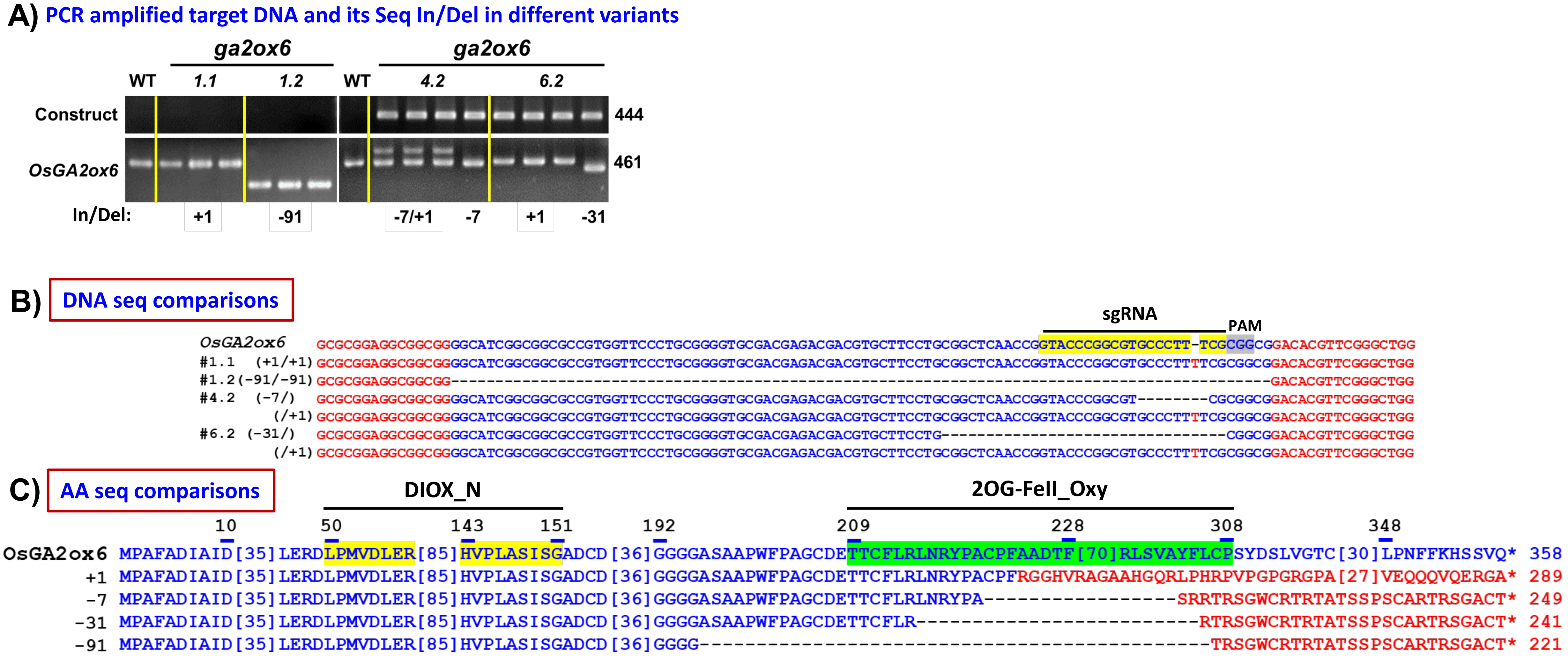

Fig S3-6. PCR validation, sequence analysis, and amino acid changes of ***osga2ox6*** mutants generated by CRISPR/Cas9

Osga2ox7

A) PCR amplified target DNA and its Seq In/Del in different variants

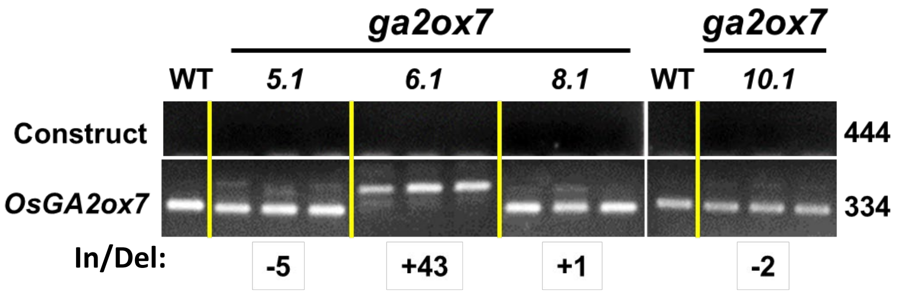

B) DNA seq comparisons

```
                                PAM                          sgRNA
OsGA2ox7          GCGACCTCGG----------------------------------------CTGGATCGAGTACCTGCTACTCTGCCT
#5.1    (-5/-5)   GCGACCTCGG---------------------------------------------TCGAGTACCTGCTACTCTGCCT
#6.1 (+43/+43)    GCGACCTCGGTGGATCGAGTACCTGCTACTCTGCCTCGCCCCGCCGCCGCCGCCGGATCGAGTACCTGCTACTCTGCCT
#8.1    (+1/+1)   GCGACCTCGG---------------------------------------TCTGGATCGAGTACCTGCTACTCTGCCT
#10.1   (-2/-2)   GCGACCTCGG------------------------------------------GGATCGAGTACCTGCTACTCTGCCT
```

C) AA seq comparisons

```
                                       DIOX_N                                  2OG-FeII_Oxy
                    10       24         101        112        179        189        287       323
OsGA2ox7  MVVLAKGELE[10]PADVRAIDLS[70]AGDLGWIEYLLLCLA[60]REDSDSILRVNHYP[90]SMVFFGGPPPGER[30]GRLADDRLCRFEN* 335
-5        MVVLAKGELE[10]PADVRAIDLS[70]AGDLG----------------RVPATLPRPRRRRRGIAVRRDVAHASLPL[82]PLQRRPRAGDLPP* 229
+43       MVVLAKGELE[10]PADVRAIDLS[70]AGDLGGSSTCYSASPPPPPLDRVPATLPRPRRRRRGIAVRRDVAHASLPL[82]PLQRRPRAGDLPP* 245
+1        MVVLAKGELE[10]PADVRAIDLS[70]AGDLG-------------LDRVPATLPRPRRRRRGIAVRRDVAHASLPL[82]PLQRRPRAGDLPP* 231
-2        MVVLAKGELE[10]PADVRAIDLS[70]AGDLG---------------DRVPATLPRPRRRRRGIAVRRDVAHASLPL[82]PLQRRPRAGDLPP* 230
```

Fig S3-7. PCR validation, sequence analysis, and amino acid changes of ***osga2ox7*** mutants generated by CRISPR/Cas9

Osga2ox8

A) PCR amplified target DNA and its Seq In/Del in different variants

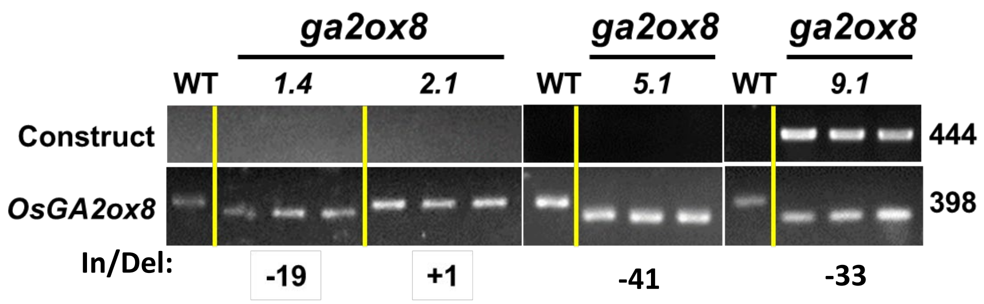

B) DNA seq comparisons

```
                              PAM      sgRNA
OsGA2ox8          AATGCCGGGCCGCAAGCTGTCATCCCGTGC-ATCGACCTGTCGGCACCGGGCGCGGCGGCGGCGGTGGC
#1.4 (-19/-19)    AATGCCGGGTCGGCA--------------------ACCTGTCGGCACCGGGCGCGGCGGCGGCGGTGGC
#2.1   (+1/+1)    AATGCCGGGCCGCAAGCTGTCATCCCGTGCAATCGACCTGTCGGCACCGGGCGCGGCGGCGGCGGTGGC
#5.1 (-41/-41)    AATGCCGGG------------------------------------------CGCGGCGGCGGCGGTGGC
#9.1 (-33/-33)    AATGCCGGGCCGCAAGCTGTCATCC----------------------------------CGGCGGTGGC
```

C) AA seq comparisons

```
                            DIOX_N                              2OG-FeII_Oxy
           10             30                48        123     129             179     185             292             331
OsGA2ox8  MVAITAPSSI[10]GANAGPQAVIPCIDLSAPGAAAAVADAC[70]AASSGGAALPA[45]GCEGSDEMLRV[100]VIYFGGPAASQRIA[31]GDNRLGPYELQHAAANDEAATKK* 353
     -33  MVAITAPSSI[10]GANAGPQAVIP-----------AVADAC[70]AASSGGAALPA[45]GCEGSDEMLRV[100]VIYFGGPAASQRIA[31]GDNRLGPYELQHAAANDEAATKK* 342
      +1  MVAITAPSSI[10]GANAGPQAVIPC------NRPVGTGRGG[70]PPPLGRRRLVR[45]AAADGGGVRGQ[100]GGVEAVGDLLRRAS[31]RLQDAPRRQPPRPLRAAARRCQR* 354
     -41  MVAITAPSSI[10]GANAG--------------------RGG[70]PPPLGRRRLVR[45]AAADGGGVRGQ[100]GGVEAVGDLLRRAS[31]RLQDAPRRQPPRPLRAAARRCQR* 340
     -19  MVAITAPSSI[10]GANAGSATCRHRARRRRWPTRAAPWGSSRRPTTASPRGSPTRWSRAPWRSSRSRTRRSSTCPAPPGPSATAARASGRTATWGGWSTSSSRPAPPRPAARRCRRR* 124
```

Fig S3-8. PCR validation, sequence analysis, and amino acid changes of ***osga2ox8*** mutants generated by CRISPR/Cas9

Osga2ox9

A) PCR amplified target DNA and its Seq In/Del in different variants

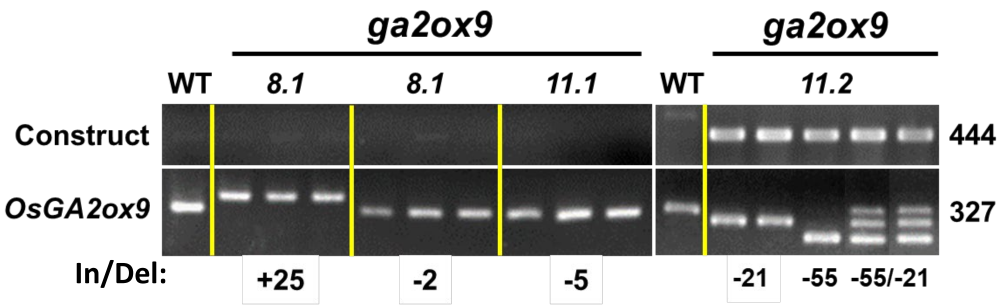

B) DNA seq comparisons

```
                                                                  PAM         sgRNA
OsGA2ox9        TGCCTGACGGCGCCGACCGGCGCCGCCGCCGCCGCCGCCGTGGGG--------------------------CAGCAGCATCAAGCCGAGGAGAGGGCG
#8.1(+25/+25)   TGCCTGACGGCGCCGACCGGCGCCGCCGCCGCCGCCGCCGTGGAGAGGGCTGGCGCATCCTGAACTCGATCTGCAGCATCAAGCCGAGGAGAGGGCG
#8.1  (-2/-2)   TGCCTGACGGCGCCGACCGGCGCCGCCGCCGCCGCCGCCGTGG--------------------------CAGCAGCATCAAGCCGAGGAGAGGGCG
#11.1 (-5/-5)   TGCCTGACGGCGCCGACCGGCGCCGCCGCCGCCGCCGCCG-----------------------------CAGCAGCATCAAGCCGAGGAGAGGGCG
#11.2 (-55/)    TGCCTGAGG------------------------------------------------------------------------AGAGGGCG
      (/-21)    TGCCTGACGGCGCCGACCGGCGCCGCCGCCGCCGCCGCCGTGG--------------------------------------AGAGGGCG
```

C) AA seq comparisons

```
                                         DIOX_N                                                 2OG-FeII_Oxy
                10          47                                 81           163              214             314                347
OsGA2ox9  MPAIADCAAD[30]PVSECELPMIDVGCLTAPTGAAAAAAVGQQHQAEERAACAA[75]AGISGKSCNYGD[40]EGCDDATCFL[90]AYFLCPSYDSPIG[25]KTGKKTGLSNFLV* 359
-21       MPAIADCAAD[30]PVSECELPMIDVGCLTAPTGAAAAAA--------ERAACAA[75]AGISGKSCNYGD[40]EGCDDATCFL[90]AYFLCPSYDSPIG[25]KTGKKTGLSNFLV* 352
-2        MPAIADCAAD[30]PVSECELPMIDVGCLTAPTGAAAAAAVAAASSRGEGGLRGG[75]RHLRQILQLRRP[90]EGLPLGRRQA[30]ERDDGEILRRLLP[30]GRRQEDGEEDWPQ* 354
-5        MPAIADCAAD[30]PVSECELPMIDVGCLTAPTGAAAAAA-AAASSRGEGGLRGG[75]RHLRQILQLRRP[90]EGLPLGRRQA[30]ERDDGEILRRLLP[30]GRRQEDGEEDWPQ* 353
-55       MPAIADCAAD[30]PVSECELPMIDVGCLTRGRPARRPSRRRPRSGGSSRW* 77
+25       MPAIADCAAD[30]PVSECELPMIDVGCLTAPTGAAAAAAERAGAS* 73
```

Fig S3-9. PCR validation, sequence analysis, and amino acid changes of ***osga2ox9*** mutants generated by CRISPR/Cas9

A) The effects of ***ga2ox3*** interact with other class I mutants#

3/478 \ Xxx	4	7	8	47	48	78	478
34**	0.0024						
37		NA					
38*			0.0247				
347***				0.0001			
348					NA		
378**						0.0068	
3478***							<0.0001

B) The effects of ***ga2ox4*** interact with other class I mutants#

4/378 \ Xxx	3	7	8	37	38	78	378
34***	0.0003						
47*		0.0226					
48			NA				
347				NA			
348					0.0356		
478***						<0.0001	
3478***							<0.0001

C) The effects of ***ga2ox7*** interact with other class I mutants#

7/348 \ Xxx	3	4	8	34	38	48	348
37	NA						
47***		<0.0001					
78***			<0.0001				
347*				0.0325			
378**					0.0083		
478						NA	
3478**							0.0095

D) The effects of ***ga2ox8*** interact with other class I mutants#

8/347 \ Xxx	3	4	7	34	37	47	347
38*	0.0182						
48		NA					
78***			0.0004				
348				0.1040			
378					NA		
478*						0.0148	
3478***							0.0003

Fig. S4 Interaction effects among Class I ***ga2ox*** mutants revealed by pairwise comparisons

RPH data from available single- and multiple-gene KO mutant combinations were compared to *ga2ox3* (A), *ga2ox4* (B), *ga2ox7* (C) and *ga2ox8* (D) mutants, respectively, using the Nonparametric Mann-Whitney test to reveal their difference levels. *P*-values (**p* < 0.05, ***p* < 0.01, ****p* < 0.001) are shown in their correspondent boxes and color-coded for additive effects (red), suppressive effects (green), no effect (gray). NA: not available
