# Supplementary material for: Systematic mutagenesis reveals dominant–minor paralog configurations in the rice GA2ox gene family
Source: Front Plant Sci. 2026 May 7;17:1813123. doi: 10.3389/fpls.2026.1813123 (PMC13190458; doi:10.3389/fpls.2026.1813123)
Supplement: Supplementary Figure S2-1 — Schematic diagram to describe the screening and selection process for gene-edited osga2oxs mutation variants. The schematic workflow shows the generation and screening of osga2oxs CRISPR/Cas9-edited rice plants. Wild-type calli were transformed, and regenerated T0 plants were analyzed by PCR and sequencing for T-DNA construct (Cnt) presence and target site mutations. Gene-edited T1 plants were classified into homozygous, heterozygous/biallelic, and non-edited categories, each with or without the Cnt. During T2 generation selection, non-edited plants and heterozygous lines carrying the Cnt were eliminated. Successive generations (T2–T6) were propagated and analyzed by PCR and sequencing to monitor allele segregation and remove the Cnt. This process yielded stable homozygous A- and B-variants, including Cnt-free lines suitable for downstream functional studies. [file DataSheet2.pdf]

Table S1. List of primers and their sequences used in this study

sgRNA for *OsGA2ox* genes knockout

Name of primer sets	Sequences
*OsGA2ox1*_sgRNA-F	5′-**GGCA**GGTGGCGAGGCAGGTGGCGA-3′
*OsGA2ox1*_sgRNA-R	5′-**AAAC**TCGCCACCTGCCTCGCCACC-3′
*OsGA2ox2*_sgRNA-F	5′-**GGCA**GCCGGCGACCACAAGTCCGG-3′
*OsGA2ox2*_sgRNA-R	5′-**AAAC**CCGGACTTGTGGTCGCCGGC-3′
*OsGA2ox3*_sgRNA-F	5′-**GGCA**GCGCGCTGGTGACGGCGGAA-3′
*OsGA2ox3*_sgRNA-R	5′-**AAAC**TTCCGCCGTCACCAGCGCGC-3′
*OsGA2ox4*_sgRNA-F	5′-**GGCA**GGAGAGGGACGCGCCGTCGA-3′
*OsGA2ox4*_sgRNA-R	5′-**AAAC**TCGACGGCGCGTCCCTCTCC-3′
*OsGA2ox5*_sgRNA-F	5′-**GGCA**GCTTCTCGCCGGAGAGCTAC-3′
*OsGA2ox5*_sgRNA-R	5′-**AAAC**GTAGCTCTCCGGCGAGAAGC-3′
*OsGA2ox6*_sgRNA-F	5′-**GGCA**GTACCCGGCGTGCCCTTTCG-3′
*OsGA2ox6*_sgRNA-R	5′-**AAAC**CGAAAGGGCACGCCGGGTAC-3′
*OsGA2ox7*_sgRNA-F	5′-**GGCA**GCAGGTACTCGATCCAGCCG-3′
*OsGA2ox7*_sgRNA-F	5′-**AAAC**CGGCTGGATCGAGTACCTGC-3′
*OsGA2ox8*_sgRNA-F	5′-**GGCA**GGTGCCGACAGGTCGATGCA-3′
*OsGA2ox8*_sgRNA-F	5′-**AAAC**GGTGCCGACAGGTCGATGCA-3′
*OsGA2ox9*_sgRNA-F	5′-**GGCA**GGCTTGATGCTGCTGCCCCA-3′
*OsGA2ox9*_sgRNA-R	5′-**AAAC**TGGGGCAGCAGCATCAAGCC-3′

For genotyping and T-DNA construct

Name of primer sets	Sequences	Size of PCR product (bp)
OsGA2ox1-CRP-1F	5′- TGACGATAGCGACGGTGGAC-3′	594
OsGA2ox1-CRP-1R	5′- TGACACGTAGGCGCAACACA-3′	
OsGA2ox2-CRP-2F	5′- TTGACGAAGAACGCGGACGGGT-3′	362
OsGA2ox2-CRP-2R	5′- AATGATTATGTGGAGGCAGTGAGGCA-3′	
OsGA2ox2-RT-2R	5′-CTATTCATGGTCGTCATCGTCC-3′	1589 (with RT-2R)
OsGA2ox3-CRP-3F	5′- CCGACGGCGATATATAATATCAG-3′	504
OsGA2ox3-CRP-3R	5′- GAAGGTAGCGCGTTGATGGAA-3′	
OsGA2ox4-CRP-4F	5′- TTGGGTTCTTCAAGGTGGTGAG-3′	488
OsGA2ox4-CRP-4R	5′- GTTCAGCCGGAGGATCTGGT-3′	
OsGA2ox5-CRP-5F	5′- CAAGCACCTGTTCGTGGAGCAGC-3′	467
OsGA2ox5-CRP-5R	5′- GCCCTCTCATCAGGATCTCTGCC-3′	
OsGA2ox6-CRP-6F	5′- TACTGCCGGTTGGTGCACATGGAC-3′	461
OsGA2ox6-CRP-6R	5′- AATCGGCGGACTGTTACCTGAAACA-3′	
OsGA2ox7-CRP-7F	5′- TGCGAGGAGCAGGGGTTCTTC-3′	334
OsGA2ox7-CRP-7R	5′- ACACCGCGAGATAGTAGCAGTAGGG-3′	
OsGA2ox8-CRP-8F	5′- TGTTCTTGCTCTGTTTCCTCACTCTGTTGG-3′	398
OsGA2ox8-CRP-8R	5′- CCGTTCGACCCGATGCTCTTGC-3′	
OsGA2ox9-CRP-9F	5′- CTACTACACGCTGCTCCGCCTCG-3′	327
OsGA2ox9-CRP-9R	5′- CTGGACTTGGCCTCGAACGGGA-3′	
F	5′- TCACACAGGAAACAGCTATGAC-3′	444
R	sgRNA-R from each target gene for the construct	
hpt-F	5′- GCTTCGATGTAGGAGGGCGTG-3′	343
hpt-R	5′- GCTCCAGTCAATGACCGCTGTTAT-3′	
