# Supplementary material for: Systematic mutagenesis reveals dominant–minor paralog configurations in the rice GA2ox gene family
Source: Front Plant Sci. 2026 May 7;17:1813123. doi: 10.3389/fpls.2026.1813123 (PMC13190458; doi:10.3389/fpls.2026.1813123)
Supplement: Supplementary Figure 3-1 — PCR validation, sequence analysis, and amino acid changes of osga2ox1 variants generated by CRISPR/Cas9. (A) PCR amplification of the OsGA2ox1 target region in wild type (WT) and representative variant lines (2.1, 5.3, and 5.4). Band sizes correspond to WT (594 bp) and insertion or deletion (In/Del) variants: +1 (595 bp), −84 (510 bp), and −42 (552 bp). (B) DNA sequence alignments of OsGA2ox1 in WT and variants. The sgRNA sequence (yellow) and PAM (grey) are highlighted. Variant alleles show an insertion (+1) or two deletions (−84, −42) at the target site, generating In/Del polymorphisms. (C) Amino acid sequence comparisons of OsGA2ox1 in WT and variants. Predicted protein products reveal altered ORFs due to In/Del-induced frameshift mutations, disrupting the conserved domains DIOX_N and 2OG-FeII_Oxy. Functional motifs (yellow and green) are either disrupted or lost, and regions with disrupted ORFs are highlighted in red. [file DataSheet3.pdf]

Class II

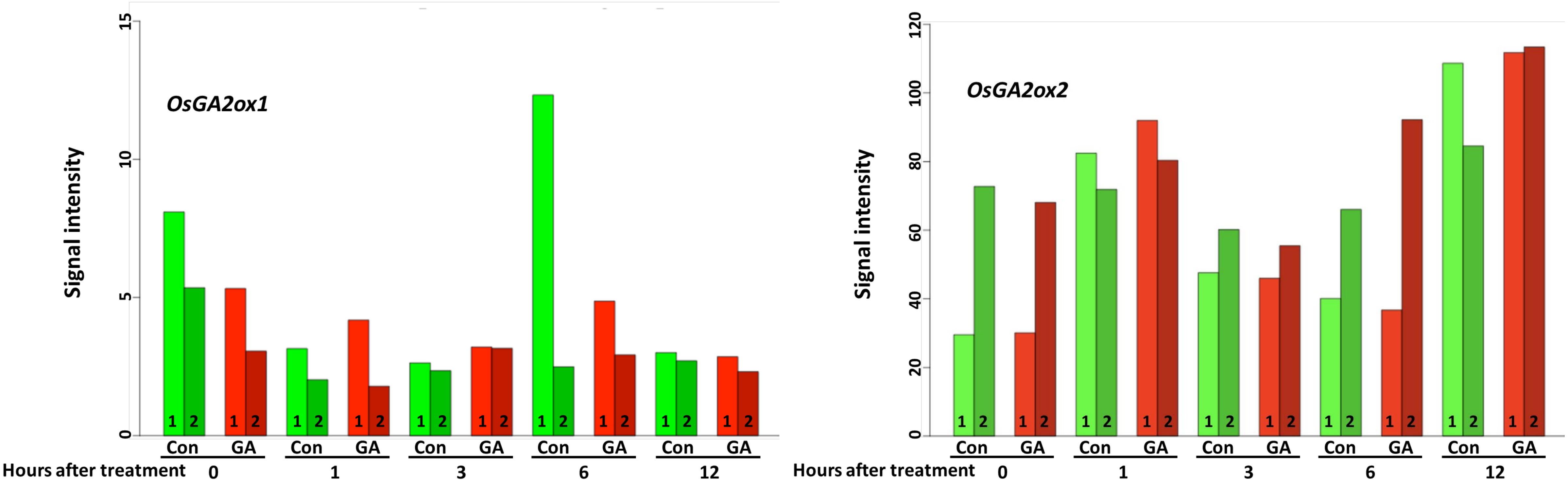

Supplementary Figure 5. Expression profiles of OsGA2ox gene family in response to GA treatment

Expression profiles of *OsGA2ox* genes from Class II (*OsGA2ox1* and *OsGA2ox2*), Class I (*OsGA2ox3*, *OsGA2ox4*, *OsGA2ox7*, and *OsGA2ox8*), and Class III (*OsGA2ox5*, *OsGA2ox6*, and *OsGA2ox9*) following GA treatment were obtained from the RiceXPro database. Seven-day-old rice seedlings (*Oryza sativa* L. japonica cv. Nipponbare) were treated with 10 µM GA_3, and shoot samples were collected at 0, 1, 3, 6, and 12 hours after treatment.

Expression levels are presented as signal intensity values. Green bars represent control samples, and red bars represent GA-treated samples. Two biological replicates are shown for each time point.

Fig S5-2

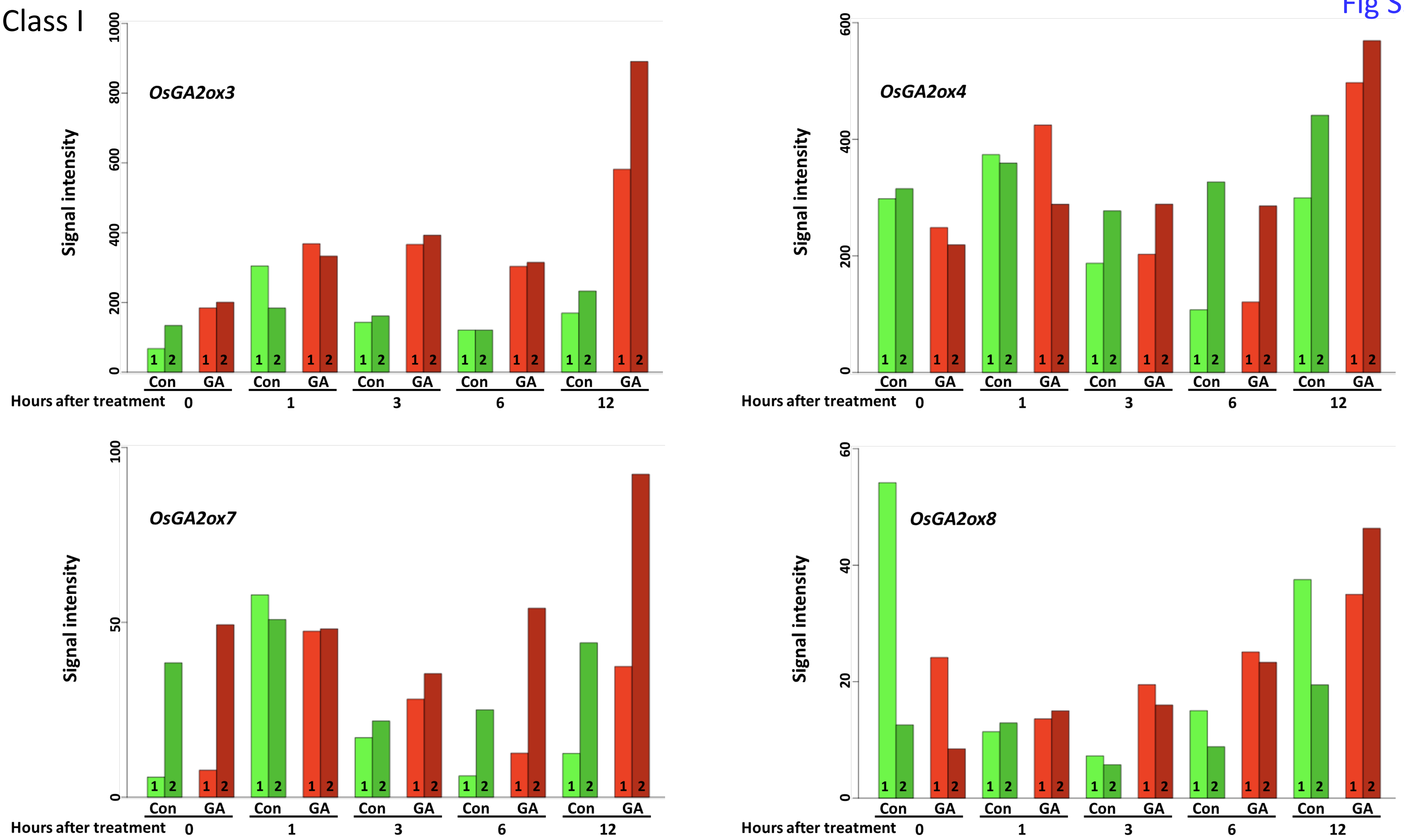
Class I
OsGA2ox3
OsGA2ox4
OsGA2ox7
OsGA2ox8
Signal intensity
0
200
400
600
800
1000
0
200
400
600
0
50
100
0
20
40
60
1 2
Con
GA
Hours after treatment
0
1
3
6
12

Fig S5-3

Class III

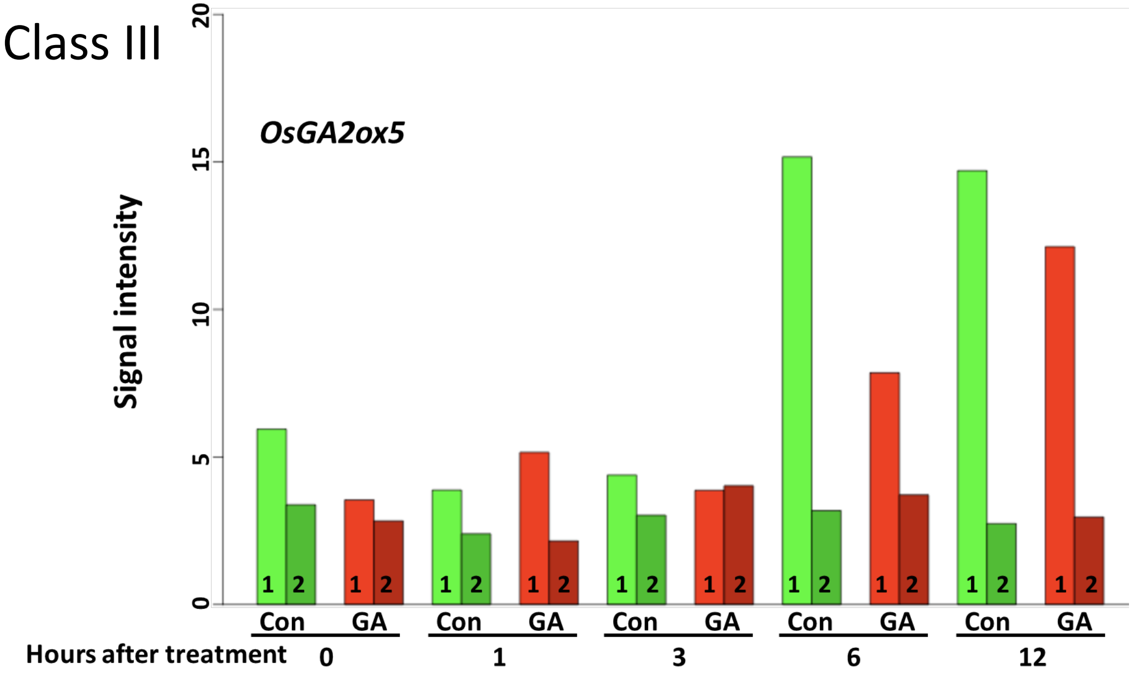

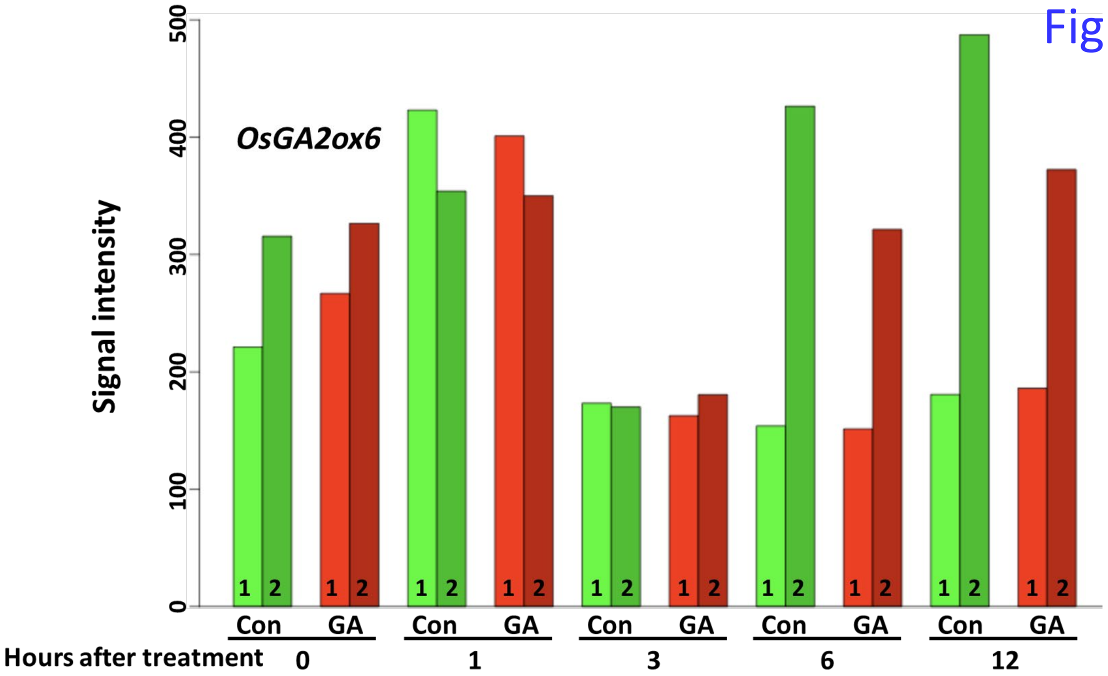

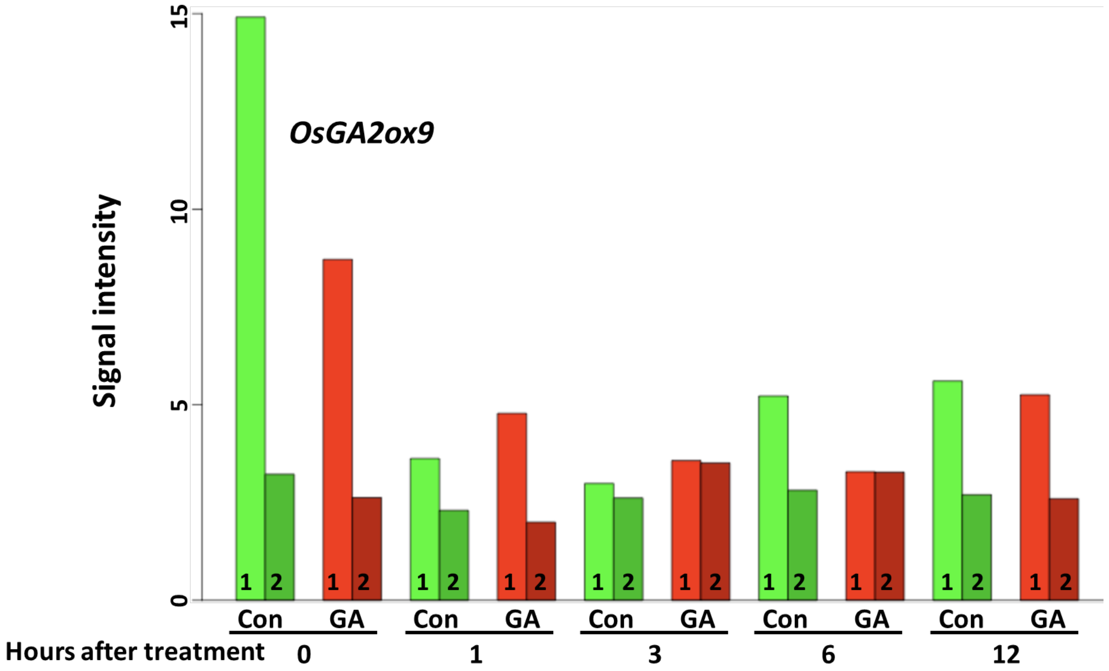

A) Analysis of Class I mutants *osga2ox3, osga2ox4, osga2ox7* and *osga2ox8*

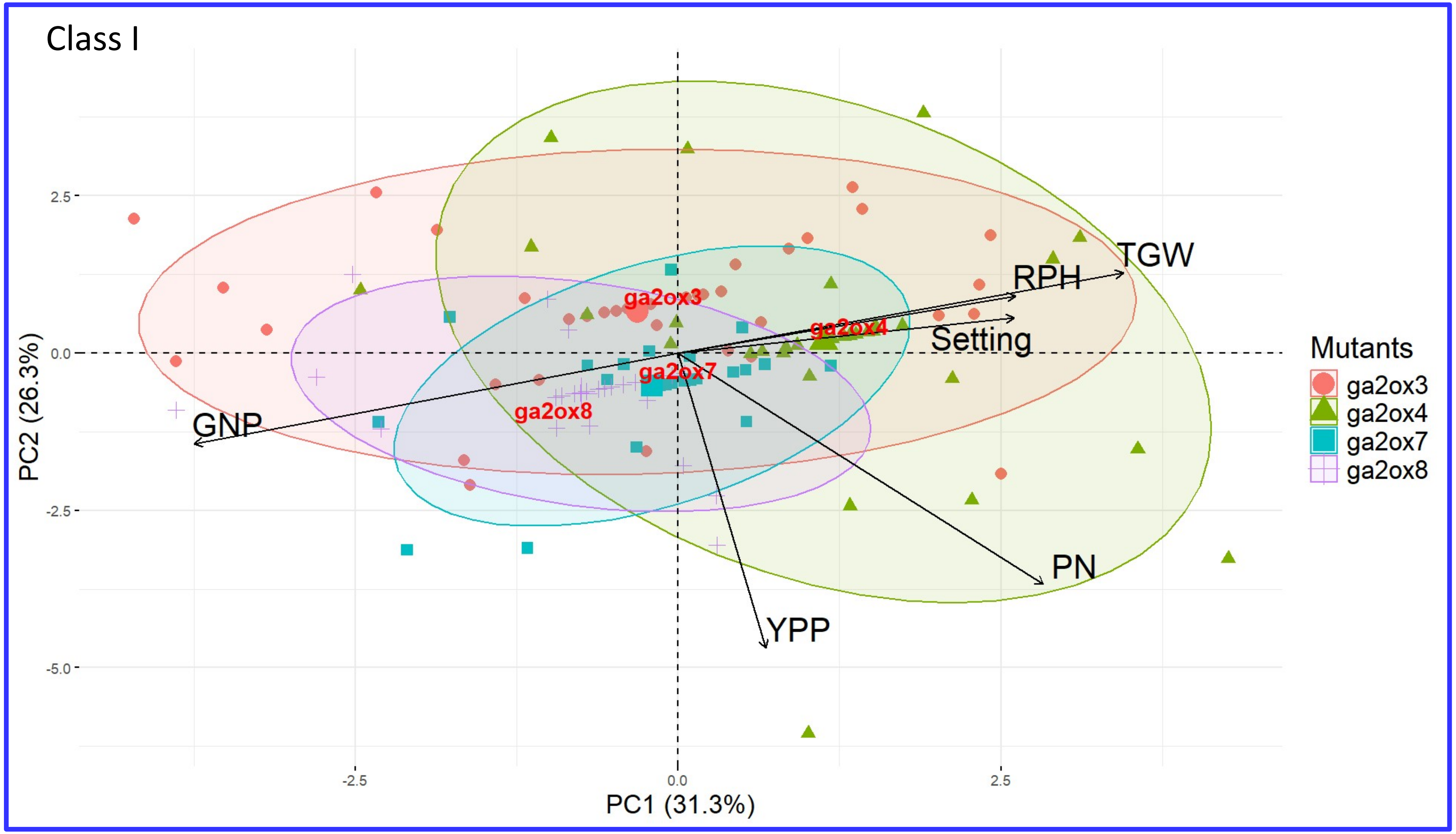

Fig S6-2

B) Analysis of Class II mutants *osga2ox1* and *osga2ox2*

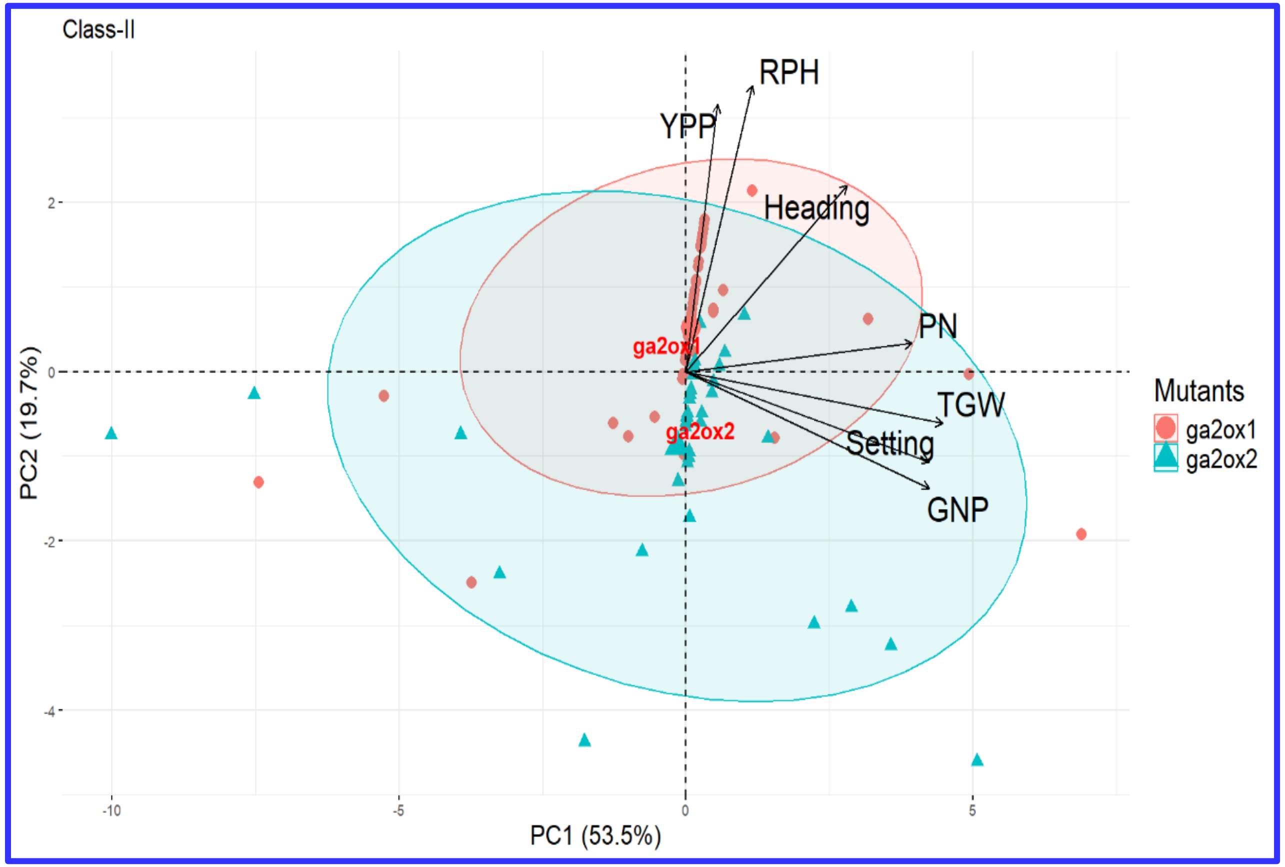

C) Analysis of Class III mutants *osga2ox5, 6* and *9*

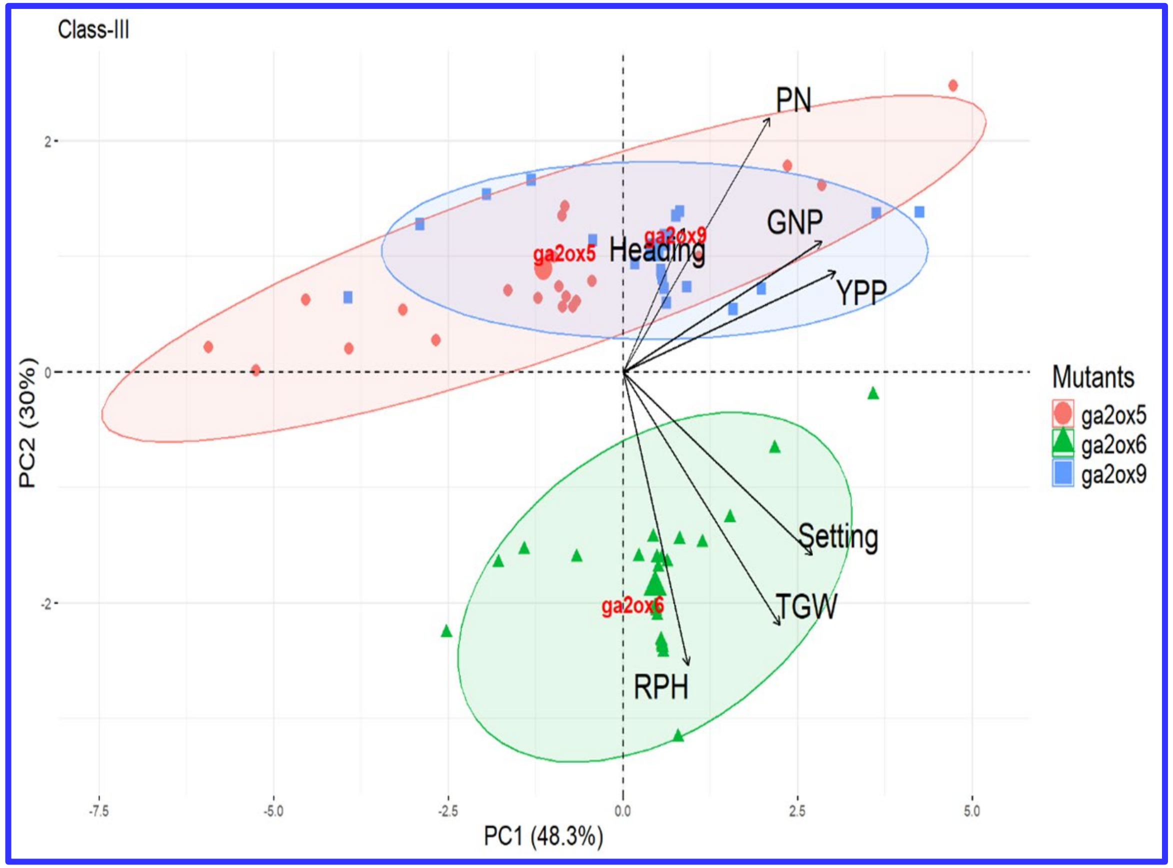

Supplementary Figure 6. Principal component analysis (PCA) of phenotypic traits in *OsGA2ox* mutant classes

Principal component analysis (PCA) of phenotypic traits was performed for (A) Class I (*osga2ox3* and *osga2ox4*, *osga2ox7* and *osga2ox8)* (B) Class II (*osga2ox1* and *osga2ox2*), and (C) Class III (*osga2ox5, osga2ox6,* and *osga2ox9*) mutants. Each point represents an individual plant, and colored symbols indicate different mutant genotypes. Ellipses represent the distribution of each genotype group.

Fig S7

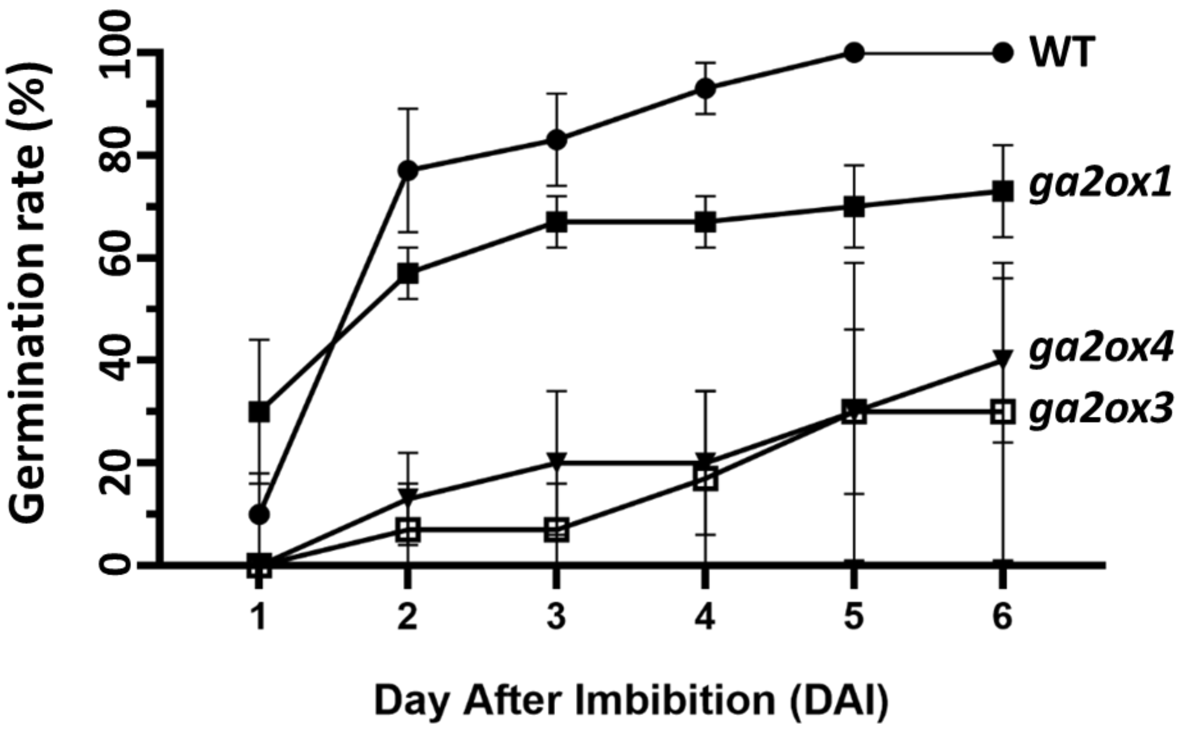

Supplementary Figure 7. Germination rate of WT and *osga2ox* mutants. Seeds from WT and *osga2ox1, osga2ox3,* and *osga2ox4* mutants were germinated on MS medium and incubated in a growth chamber at 28 °C under a 16 h light / 8 h dark photoperiod. Germination rates were recorded over 6 days after imbibition (DAI). Data represent the mean ± SD from three independent experiments, each consisting of 10 seeds (total n = 30 per genotype).
