# Supplementary material for: Systematic mutagenesis reveals dominant–minor paralog configurations in the rice GA2ox gene family
Source: Front Plant Sci. 2026 May 7;17:1813123. doi: 10.3389/fpls.2026.1813123 (PMC13190458; doi:10.3389/fpls.2026.1813123)
Supplement: Supplementary Figure S4 — Interaction effects among Class I ga2ox mutants revealed by pairwise comparisons. Relative plant height (RPH) data from available single- and multiple-gene knockout combinations were compared with ga2ox3 (A), ga2ox4 (B), ga2ox7 (C), and ga2ox8 (D) mutants using the nonparametric Mann–Whitney test. P-values (*p < 0.05, **p < 0.01, ***p < 0.001) are shown in the corresponding boxes and color-coded to indicate additive effects (red), suppressive effects (green), or no detectable effects (gray). NA indicates data not available. [file DataSheet4.pdf]

Table S2. Factorial linear model analysis of pairwise genetic interactions among *OsGA2ox* class I genes based on relative plant height (RPH).

Gene combination	Interaction term	Estimate (β)	95% CI	P-value	Interpretation
ga2ox3/4	*OsGA2ox3 : OsGA2ox4*	+6.80	2.58 to 11.02	0.002	Positive interaction
ga2ox3/8	*OsGA2ox3 : OsGA2ox8*	+3.48	−1.22 to 8.19	0.145	Not significant
ga2ox4/7	*OsGA2ox4 : OsGA2ox7*	−11.71	−15.44 to −7.98	<0.001	Negative interaction
ga2ox7/8	*OsGA2ox7 : OsGA2ox8*	−9.52	−13.48 to −5.56	<0.001	Negative interaction

Interaction terms (β) represent deviation from additive effects. Positive values indicate synergistic (greater-than-additive) interactions, whereas negative values indicate suppressive (less-than-additive) interactions. P-values correspond to the significance of the interaction term.
